# TokenUNet: A new case for transformers integration in efficient and interpretable 3D UNets for brain imaging segmentation

**DOI:** 10.1371/journal.pone.0354511

**Published:** 2026-08-05

**Authors:** Louis Fabrice Tshimanga, Andrea Zanola, Federico Del Pup, Manfredo Atzori

**Affiliations:** 1 Department of Neuroscience, University of Padua, Padua, Italy; 2 Padova Neuroscience Center, University of Padua, Padua, Italy; 3 Department of Information Engineering, University of Padua, Padua, Italy; 4 Information Systems Institute, University of Applied Sciences Western Switzerland (HES-SO Valais), Sierre, Switzerland; University of Alberta, CANADA

## Abstract

We present TokenUNet, adopting the TokenLearner and TokenFuser modules to encase Transformers into UNets. While Transformers enable expressive global interactions among input elements in medical imaging, computational challenges hinder their deployment on common hardware. Models like (Swin)UNETR exemplify the integration of (Swin)Transformer encoders into UNets, tokenizing inputs into small subvolumes (8^3^ voxels). The Transformer attention mechanism scales quadratically with the number of tokens, which is tied to the cubic scaling of 3D input resolution. This work reconsiders the role of convolution and attention, introducing TokenUNets, a family of 3D segmentation models better suited to constrained computational environments and time frames. To mitigate computational demands, our approach maintains the convolutional encoder of UNet-like models, and applies TokenLearner to 3D feature maps. This module pools a preset number of tokens from local and global structures, decoupling token number and input size. Our results on the BraTS challenge dataset for glioma segmentation show this tokenization effectively encodes task-relevant information, yielding naturally interpretable attention maps. The memory footprint, computation times at inference, and parameter counts of our heaviest model are reduced to 38%, 10%, and 17% of the SwinUNETR values, with statistically equivalent Dice score performance, for nnunetv2 5-fold cross-validation. This work opens the way to more efficient training in computationally restrained contexts, such as 3D medical imaging. Easing model optimization, fine-tuning, and transfer-learning in limited hardware settings can accelerate and diversify the development of approaches, for the benefit of the research community.

## Introduction

Discerning the types of healthy and pathological tissues in an organ is a complex task. It requires knowledge of local and global normative patterns, variability across large control populations, as well as recurring or unique forms of health problems. We introduce TokenUNet and apply it to the problem of brain tumor segmentation. The data for this task present many of the hurdles in bioimaging: they are multimodal images, they show an organ with complex morphology and equally complex pathological anomalies; they are large files, usually expensive to label. TokenUNet is a convolutional and attentional neural network. It respectively addresses local and global pattern recognition, and reaches top-level accuracy with low time and compute costs, compared to state-of-the-art (SOTA) architectures for 3D bioimage segmentation. Deep neural networks have long been dominating the tasks of classification and segmentation in medical imaging [1], as well as the prediction of diagnosis or hospital admission based on free text reports [2], to name a few [3,4]. In this context, the Brain Tumor Segmentation (BraTS) Challenge [5] has been pivotal for measuring the impact and transferability of deep learning techniques in the medical field. The challenge consists in correctly classifying 3 types of tumor and lesion tissues in 3D multimodal MRI scans of subjects affected by glioma. The winning algorithms of the BraTS challenge are typically successful in other tissue and organ segmentation tasks [6], on datasets coming from different systems of the human body. For this reason, the BraTS leaderboard is informative of medical deep learning trends. One such trend is the dominance of convolutional models derived from UNet [7] across other medical imaging applications [8]. A second major trend is the progressive introduction [9]- [10] of Transformer [11] architectures, with their computation-heavy, expressive attention layers. Both convolution and the attention mechanism have specific feature detection properties that may be relevant to the BraTS challenge. Tissues tied to tumor growth have geometrical, physical and physiological properties that set them apart from the surrounding healthy tissues. Structures appear with different contrasts across scan modalities, in varying shapes and sizes depending on subject and tumor growth stage. Recognizing such features requires both a notion of the expected variability in healthy tissues with regards to the many anatomical structures, and a characterization of how the unhealthy tissues themselves can appear. Convolutional models excel in detecting learned local patterns, regardless of their position in the input image. However, features are aggregated only locally, thus long-range correlations can be missed. Attention models like Transformer, instead, evaluate all pairwise interactions between input tokens regardless of distance, which is computationally burdensome and possibly wasteful. Moreover, Transformers need to learn spatial biases from scratch. Hybrid models such as SwinUNETR [12], where attention encoders and convolutional decoders are concatenated in a UNet fashion, try to complement both types of operation. Despite the effectiveness of SwinUNETR, several research questions remain unanswered. It is unclear if the performance gains over fully-convolutional models are consistent across the board of 3D bioimages [13]. If so, it is yet undetermined how much these improvements depend on the attentional mechanism, rather than parameter counts, FLOPs or other differences. Drivers of performance and trade-offs are relatively unexplored, while computational requirements for both training and inference grow. Consequently, both testing theories on legacy models and incremental development become more expensive in terms of time, energy, economic resources. As a step in new directions, in this paper we propose a novel integration of convolutions and attention, encasing small Transformers between the UNet encoder and decoder, similarly to TransUNet [14]. However, we depart from the straightforward tokenization of inputs or feature maps, and repurpose TokenLearner and TokenFuser [15]. Our contributions are twofold:

- we reaffirm the case for using both efficient convolutional encoders, and Transformers in UNet
- we propose a tokenization with emergent semantic properties that naturally lends itself to mechanistic interpretability

By means of TokenLearner and TokenFuser, TokenUNet cuts the time and memory requirements for a Transformer to process a 3D image. Fixing the number of tokens processed by the Transformer, we decouple it from the large input size typical of the domain. Moreover, the modules generate and use easily inspectable attention maps that open an interpretable window onto the model “black box.” After training, these spatial attention maps show emerging alingment to output labels. We compare performance gains of each modification of a template UNet architecture, until completing a TokenUNet model. As a result, we challenge the idea that Transformer encoders or large parameter counts yield the most returns in segmentation accuracy. When the last encoder feature map is fed to TokenLearner, it classifies each of *p* pixels (or voxels) as more or less relevant to a set of *N* abstract classes, with N≪p. This process yields *N* spatial attention maps, and *N* token embeddings are pooled from feature maps according to attention scores. Token embeddings at this stage can be fed to any token processing architecture, such as Transformers (even pre-trained with self-supervision) and MLP-Mixers [16]. The TokenFuser module brings back token information to 2/3D space, for the decoder. Our results confirm how TokenLearner and TokenFuser allow to integrate Transformers into virtually any 3D CNN autoencoder, and how the integration improves simple and efficient UNets, topping performances of slower and memory-heavier SwinUNETR, with easier requirements. The effectiveness and viability of TokenLearner and TokenFuser in reducing memory footprint of training and inference could allow further developments on common hardware available to researchers. The method is naturally interpretable, thanks to its attention maps that encode the location and impact of voxels for the neural network output. The information bottleneck [17] embodied by the tokenization and detokenization may [18] nudge the network towards better representations, which leaves the possibility of better adaptation of Transformers in this framework.

### Related works

UNet is a Convolutional Neural Network (CNN) autoencoder, named after its “U”-shaped structure. The descending curve of the “U” shape is the encoder, that processes tensors progressively longer in the channel dimension, smaller in the spatial dimensions. Convolutional layers compare neighborhoods of voxels to specific intensity patterns (kernels), thus encoding how small structures are distributed in space. By reducing the resolution and size of data along the encoder (downsampling), kernels of a fixed size can uncover patterns over larger neighborhoods of the input. The ascending curve of the “U” shape refers to the decoder. Each layers of the decoder combines the output from the previous decoder layer with the output from its corresponding encoder layer, with equal resolution and size, and then upsamples the result. A decoder layer thus combines semantically enriched data from the previous decoder layer, with geometrically correct data from the corresponding encoder layer, more similar to the original input. Many UNet-like architectures build on these effective principles, adding specific modifications. In contrast, the nnU-Net (“No new net”) framework [6,13] focuses on inferring proper training and architecture hyperparameters from a dataset. Once the dataset footprint is determined, a tailored sized UNet is trained, without over-engineering new architectures with the risk of overfitting a dataset. The framework has been applied successfully to the BraTS challenge, among an array comprising 23 datasets and 53 segmentation tasks of varying shapes (2D and 3D), object scales (cells to organs) and acquisition modalities. Nonetheless, the widespread success of Transformer models outside language-based domains (e.g., Vision Transformers (ViTs) [19]) has prompted experiments with new architectures in biomedicine [20,21], including the BraTS challenge. One appealing feature of Transformers is the all-to-all information exchange between input elements, whereas CNN kernels are locally constrained and may reach a global range only when stacked. Since data may show long-range dependencies, the global interactions allowed by the attention mechanism may be better suited to encode such relationships. In order to employ Transformer encoders in 3D vision, the input is usually divided into fixed size, non-overlapping cubic patches of voxels. Each patch is flattened into an array of voxels and linearly projected into a vector of dimension *d*, called token embedding. This tokenization process can be applied either to intermediate convolutional feature maps [14], or to the original input scan [22], bypassing the convolutional encoder typical of UNet. Token embeddings are fed to Transformer blocks, where they are further mapped and interpolated with one another. The main strength of the Transformer block is also a potential hindrance. The attention mechanism computes all pairwise comparisons between token embeddings. In particular, a Self-Attention head computation on a set *X* of *N* tokens of dimension *d* is defined as:


$$\begin{array}{c} Z=\text{SA}(X)=\text{softmax}\left(\frac{XW_{Q}W_{K}^{T}X^{T}}{\sqrt{d}}\right)XW_{V}, \end{array}$$ (1)

with projection matrices $W_{Q},W_{K},W_{V}\in {\mathbb{R}}^{d\times d}$, and it has *O*(*N*^2^) computational complexity [23]. For 3D images, the number of tokens itself grows with the cube of patch resolution. As an example, doubling resolution or side incurs in an 8-fold (2^3^) increase in patches and tokens, and a 64-fold (8^2^) increase in token comparisons. This complexity hinders the widespread training and development of Transformer encoders for 3D biomedical images with common hardware, namely CPUs and single GPUs. The result is a reduced pool of laboratories able to reproduce and build on SOTA models.

Several lines of research have emerged to address the quadratic computational complexity of the original Transformer attention mechanism. One such trajectory focuses on optimizing the operation at the hardware level, implementing smart, I/O-aware kernels that compute exact attention with significantly higher memory efficiency, such as FlashAttention [24]. Another major line of work seeks to fundamentally reduce the number of computed token interactions. This is achieved either explicitly, through sparse or local attention patterns like those used in Longformer [25] and BigBird [26], or implicitly, by approximating the dense attention matrices using low-rank proxies, as seen in Linformer [27] and Performer [28]. In the medical imaging domain, architectures have adopted various structural strategies to mitigate this computational burden [29]. The TransUNet model [30] partially addresses the problem by extracting tokens only from the low-resolution bottleneck feature maps of a standard convolutional encoder. Conversely, architectures like SwinUNETR [12] bypass the convolutional encoder entirely. Building upon the Swin Transformer [31], SwinUNETR alleviates the attention burden by hierarchically merging neighboring tokens to progressively reduce the sequence length. The process is analogous to average pooling in CNNs. Our proposed TokenUNet aligns conceptually with the philosophy of sequence length reduction (see also [32], that renounces to convolutions altogether). However, rather than redefining the attention mechanism itself or relying on progressive hierarchical merging, our approach uses tokenizer modules to prescribe a fixed, highly compressed number of informative tokens to start with.. By applying this severe compression at the bottleneck, the computational cost of our attention block remains minimal and strictly independent of the original input resolution or volume size. Furthermore, this spatial tokenization inherently addresses the demand for clinical explainability. Unlike popular post-hoc methods such as Grad-CAM [33], which rely on gradient flows to provide retrospective and sometimes unfaithful [34]- [35] approximations of feature importance, our tokenizer modules yield spatial attention maps directly as a functional byproduct of the forward pass. This intrinsic interpretability ensures that the visualized attention accurately and transparently reflects the model’s actual decision-making focus [36]. Regarding the BraTS challenge and its trends, later editions have seen the rise of model ensembling, data augmentations with synthetic data [37], and clinically-aligned post-processing as means to set new SOTA [38].

## Materials and methods

This section describes the data, architecture choices and training setting of our experiments. We first develop an effective UNet variant, identified as NoTokenUNet, based on the observation that downsampling-upsampling is necessary for speed of computation, decrease of memory footprint, and performance metrics, while concatenating skip-connections can be switched to additive skip-connections with no loss of accuracy and relatively decreasing memory usage. We then evaluate TokenLearner and TokenFuser as bottlenecks. Incorporating them constitutes the TokenUNet, specifically without Transformer. Finally, we encase a small Transformer encoder or MLP-Mixer between the two Token modules. It is important to note that other token processing layers may be included for new TokenUNet variants. Our architectures are compared to a vanilla UNet and a SwinUNETR implemented according to published settings, on a 5-fold Cross Validation with 100 epochs per fold (sufficient to stabilize the loss values) using a common fold segregation and training setting based on the nnunetv2 library application to the dataset [13].

### Data

The dataset is the FeTS 2022 Challenge Dataset, a subset of the BraTS Continuous Evaluation Challenge Dataset, comprising 1251 subjects with radiographically appearing glioblastoma. Brain scans are collected in routine clinical acquisitions from several institutions, pre-operation, with multi-parametric Magnetic Resonance Imaging (mpMRI). For each subject, 4 modalities are available, from the MRI sequences: T1-weighted, T1-weighted Gadolinium post-contrast enhanced, T2-weighted, and T2-FLAIR. The original sequences vary in slice thickness and axial acquisition, but all images are resized and resampled to 1mm^3^ voxel size, and cropped or padded to 128×128×128 during training. Ground truth annotations of the tumor sub-regions are from expert neuroradiologists. Tumor labels are WT (whole tumor), TC (tumor core), and AT (active tumor). Denominations refer to tissue properties and appearance in the available modalities. Further information can be found at the Challenges sites (https://www.synapse.org/Synapse:syn28546456/wiki/617093, and https://www.synapse.org/Synapse:syn27046444/wiki/616571) and in [39].

### Architectures and modules

#### UNet.

The UNet architecture, introduced in [40], is originally a 2D CNN inspired by Fully Convolutional Neural Networks, with a contracting path or encoder, and an expansive path, or decoder. Each encoder block consists of a convolutional layer with 3×3 spatial kernels and doubling channel length, a nonlinear activation (ReLU), and a max pooling for downsampling feature maps. Each decoder block is instead comprised by a transposed convolution with 2×2 spatial kernels and halving channel length, the skip-connection concatenating the encoder feature map of the same spatial resolution, followed by two rounds of convolutional layer with 3×3 spatial kernels and nonlinear activation. UNet-like architectures in general maintain the original distinctions in contracting path and expansive path, however downsampling, upsampling, skip-connections and configurations of blocks may vary from case to case, and integrate several modifications from other innovations in computer vision. The strengths of the baseline UNet should be highlighted. First, downsampling then upsampling along the spatial dimensions allows to reduce the memory footprint and the time required for each convolutional layer to process its input, compared to isotropic networks. Encoder layers that double the feature size and halve the resolution over 3 spatial dimensions perform a 4-fold reduction of tensor “volumes.” Second, the larger number of layers and parameters allowed makes the networks more expressive, although requiring more compute. In practice the lack of optimized CUDA operations that would theoretically require less parameters and FLOPS (e.g., depthwise separable convolutions in 3D), end up in more compute time, while underfitting. Thus, we did not alter convolutional operations to improve the UNet blueprint. In this work, our baseline UNet is a stack of residual blocks [41] where the residual function is a sequence of instance normalization layers (IN), LeakyReLU activations (ACT), and maintains a constant 3×3×3 size for all 3D convolutional kernels (CONV), in the order [IN, ACT, CONV, IN, ACT, CONV], with the inner convolution doubling the number of channels. Feature map resolution between stages is adjusted by trilinear upsampling or average pooling, channel length is adjusted by pointwise convolution. These adjustments work also on skip connections inside, between residual blocks, stages, and from encoder to decoder. Moreover, all skip connections are additive rather than concatenating. This change allows for approximately halving the memory footprint, speed, and parameter count of the decoder, with no loss of expressiveness and little overhead, at least at inference.

#### TokenLearner and TokenFuser.

The TokenUNet variants encase a TokenLearner and TokenFuser module between the CNN encoder and the MLP classifier, as shown in 1. TokenLearner is based on a simple idea: distant pixels or patches of an image can share the same features, constitute the same structure, or belong to the same abstract class. On this premise, it is possible to select such pixels and pool the original image to aggregate only this specific information, regardless of distance and dismissing irrelevant noise in the vicinity. Instead using 1 token per patch of neighboring pixels (voxels), 1 token can represent a size-independent set of variously akin pixels. In practice, the original TokenLearner employs a Multi-Layer Perceptron (MLP) as a nonlinear map to evaluate each image element’s pertinence to *N* non exclusive classes, based on its vector of features. Our versions only uses a Linear layer, which can be viewed as a pointwise, 1×1×1 convolution, followed by a sigmoid activation, making it a nonlinear map. The soft classification into *N* categories yields *N* spatial attention masks: each pixel (voxel, patch) location has its sigmoid attention score, serving as a class logit. With 3D image feature map *X* of height *H*, width *W*, depth *D*, nonlinear map MLP_TL_, and spatial attention masks collected in $A_{N}$:


$$\begin{array}{c} X\in {\mathbb{R}}^{F\times H\times W\times D}\\ {\text{MLP}}_{\text{TL}}:{\mathbb{R}}^{F...}\mapsto {\mathbb{R}}^{N...}\\ A_{N}=\underset{H,W,D}{\text{softmax}}\left({\text{MLP}}_{\text{TL}}(X)\right),\;\text{with }A_{N}\in {\mathbb{R}}^{N\times H\times W\times D}. \end{array}$$ (2)

The *N* sets of attention scores act as weights for *N* global average poolings of the image tensor, yielding *N* global vectors, each focusing on a semantic group of pixels. Using a simplified Einstein notation:


$$\begin{array}{c} T=A_{N}X,\;t_{nf}=a_{nhwd}x_{fhwd},\;\text{with }T\in {\mathbb{R}}^{N\times F}. \end{array}$$ (3)

Equivalently, one can see each spatial attention mask $A_{n}$ with n∈{1,2,...,N} broadcast over all *F* feature channels with operator $\beta_{F}(\cdot )$, followed by sum reduction of the spatial dimensions, thus $t_{n}=\beta_{F}(A_{n})X$.

The TokenFuser module is introduced to transform the *N* tokens back into the original shape and resolution of the input. One could perform “unpooling” of the tokens, by an outer product between tokens and original attention masks, effectively tiling the token for each *n*-th class of *N* into the original tensor shape, and then re-weighting according to the original contribution of a pixel for class *n*. Instead of this parameter-free unpooling, in TokenFuser a new nonlinear projection outputs new spatial attention masks. Crucial for the effectiveness of TokenFuser, a mixing matrix *M* linearly combines global tokens before unpooling.


$$\begin{array}{c} {\text{MLP}}_{\text{TF}}:{\mathbb{R}}^{F...}\mapsto {\mathbb{R}}^{N...}\\ B_{N}={\text{MLP}}_{\text{TF}}(X),\;\text{with }B_{N}\in {\mathbb{R}}^{N\times H\times W\times D}\\ Y=B_{N}MT,\;y_{fhwd}=b_{nhwd}m_{nn}t_{nf},\;Y\in {\mathbb{R}}^{F\times H\times W\times D}\\ X\leftarrow X+Y \end{array}$$ (4)

In this work *N*: = 8,32, noting that the semantically relevant elements for segmentation are at least 3 (the number of ground truth labels).

#### Token processing.

The tokens extracted by TokenLearner are processed by a typical Transformer architecture with 2−8 (our usual vs “Long” variants) Transformer encoder blocks, each made of 2 residual blocks: MultiHead Self-Attention (MHA), and MLP as Feed-Forward Network (FFN), or by an MLP-Mixer that uses another FFN applied along token indices, substituting MHA. The model size determines the width of matrices in the MHA and FFN modules, repeating the following structure for every *i*-th block:


$$\begin{array}{c} X_{\text{MHA},i}\leftarrow X_{\text{FFN},i-1}+\text{MHA}(\text{LN}(X_{\text{FFN},i-1}))\\ X_{\text{FFN},i}\leftarrow X_{\text{MHA},i}+\text{FFN}(\text{LN}(X_{\text{MHA},i})), \end{array}$$ (5)

with


$$\begin{array}{c} \text{FFN}:\text{MLP}(X)=(\phi (XW_{1}))W_{2}\\ \text{MHA}:\left(\underset{h\in H}{\text{concat}}\left[{\text{SA}}_{h}\right]\right)W_{O}\\ \text{SA}:\text{softmax}\left(\frac{XW_{Q}W_{K}^{T}X^{T}}{\sqrt{d/h}}\right)W_{V}\\ \text{LN}:\gamma \left(\frac{X-\mu_{X}}{\sigma_{X}}\right)+\beta . \end{array}$$ (6)

In the present models, for every MLP, $W_{1}\in {\mathbb{R}}^{d\times 4d},W_{2}\in {\mathbb{R}}^{4d\times d}$, and ϕ(·) is the ReLU nonlinearity rather than the costly GELU [42]; for every SA head and MHA module respectively, $W_{Q},W_{K},W_{V}\in {\mathbb{R}}^{d/h\times d/h}$ and $W_{O}\in {\mathbb{R}}^{d\times d}$, with *d* model dimension (the number of features for tokens, thus *d* = *F* = 256 of the convolutional encoder) and *h* = 8 number of heads.

**Fig 1:**
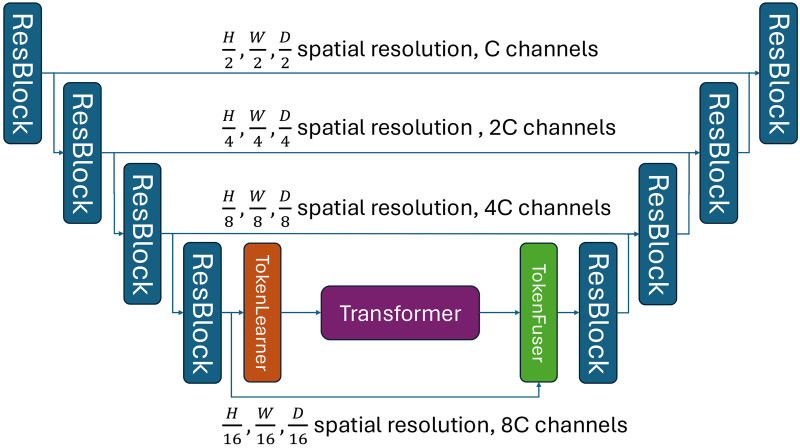
Exemplar architecture of TokenUNet, with 4 single-block stages, and including the encased Transformer.

**Fig 2:**
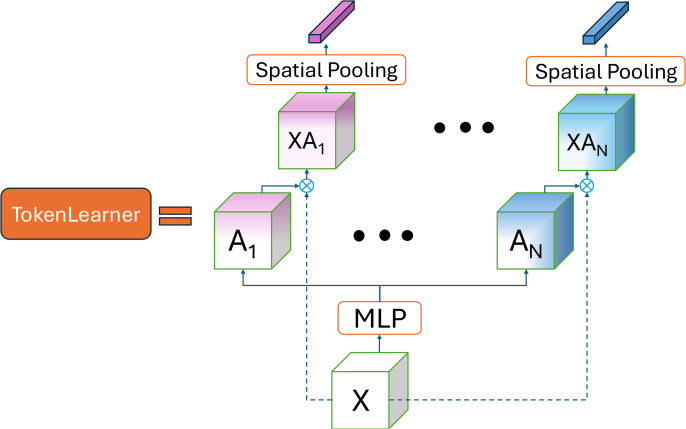
The last encoded cube is fed to TokenLearner and transformed into N different pooled vectors that encode semantic information from all pertinent locations. These vectors eventually serve as tokens in the Transformer.

**Fig 3:**
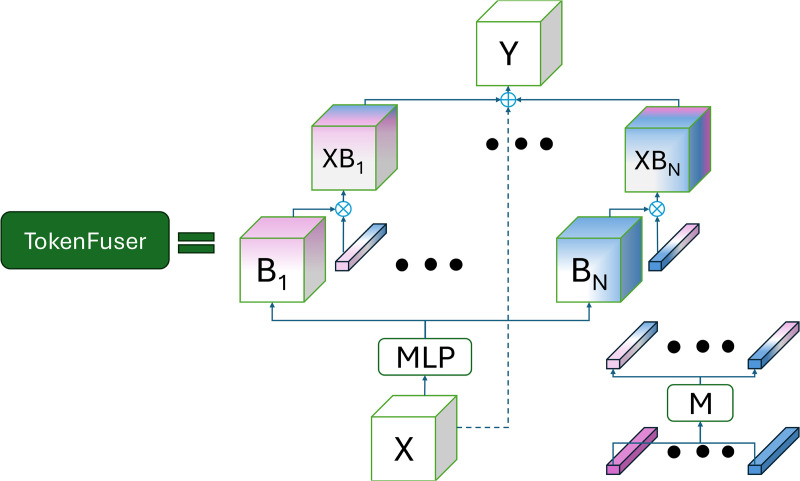
TokenFuser recreates N spatial masks for tokens, that are linearly mixed, then broadcast over the masks and finally summed to the last encoded cube, that will be decoded by the ascending UNet path.

Fig 1 shows the overall architecture of a Transformer in a TokenUNet, while Figs 2 and 3 illustrate the crucial tensor-to-token embedding transformations.

Table 1 shows the different combinations of components that characterize the plain UNet, the TokenUNets that do not use sequence models to further process token embeddings, and those that do. Table 2 summarizes the different variants and sizes, with their respective parameter counts (M for 10^6^ parameters).

**Table 1:** Components and incremental additions from UNet to TokenUNet.

	NoTokenUNet	*N*TokenUNet	*N*AttnTokenUNet	*N*MLPTokenUNet
**Tokenizer**		✓	✓	✓
**Transformer**			✓	
**MLP-Mixer**				✓
**Detokenizer**		✓	✓	✓
**Pre-trained weights**				

**Table 2:** Trainable parameter counts (in millions).

Model	NoToken	8Token	8Attn	8MLP	32Token	32Attn	32LAttn	32MLP	Swin UNETR
**Parameters (M)**	1.67	1.69	1.95	1.82	1.69	1.95	2.74	1.86	15.71

### Training

hlWe trained and tested our models following the nnUNet framework [6]- [13], with a 5-fold Cross Validation over the BraTS training set, since the Challenge validation data are unlabeled. The subjects were randomly divided into 5 folds, and this division was maintained across experiments with different model sizes and weight initialization schemes. Each fold training ran for 100 epochs. The samples were augmented by random transformations: spatial augmentations (rotation and scaling, mirroring), Gaussian noise/blur, intensity augmentations (brightness, contrast, gamma changes), and low-resolution simulation. Refer to the official repository for the respective default parameters. The learning objective minimized an averaged Dice and cross-entropy loss, without deep supervision, in order to evaluate TokenLearner and TokenFuser without nudging the alignment of spatial attention and labels. The models were all optimized with SGD with Nesterov momentum of value 0.99 [43]- [44] and learning rate of $1\cdot 10^{-2}$. All learning rates were reduced with a polynomial annealing schedule [45]. The actual batch size was set to 2, and 1 for the SwinUNETR model only, to alleviate the memory footprint. During training, patches of size 128×128×128, randomly extracted from the images, were fed to the model, once per subject; during evaluation, sliding window inference on 128×128×128 windows was integrated over the whole 3D brain scans. The final performance was measured in terms of Dice score, precision, and recall. Experiments were implemented in PyTorch (version 2.0.1) (https://pytorch.org/), MONAI (version 1.2.0) (https://monai.io), and nnunetv2 [13]. Experiments were run on a computing node with 64 Intel(R) Xeon(R) Silver 4314 CPU @ 2.40GHz, connected to a NVIDIA A30 Tensor Core GPU with 24 GB of GPU memory. The code to replicate our results will be made available at https://github.com/MedMaxLab/tokenunet upon publication.

## Results

The results in this section resume the parameter and memory footprint of TokenUNets compared to nnUNet, NoTokenUNet and SwinUNETR, as well as the average Dice score performance of model variants, and the visualization of TokenLearner and TokenFuser outputs.

### Performance metrics

**Fig 4:**
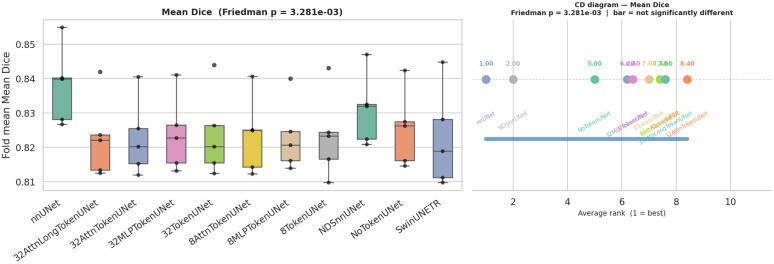
Average label Dice score, boxplots and ranking. NDSnnUNet is the nnUNet architecture trained without deep supervision.

**Fig 5:**
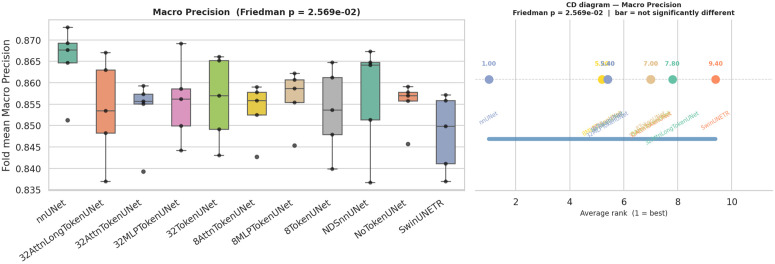
Total label precision score, boxplots and ranking. NDSnnUNet is the nnUNet architecture trained without deep supervision.

**Fig 6:**
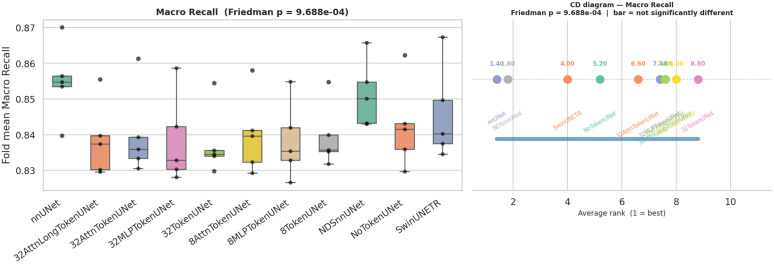
Total label recall score, boxplots and ranking. NDSnnUNet is the nnUNet architecture trained without deep supervision.

In terms of precision, recall and Dice score by label and on aggregate, TokenUNet variants were statistically equivalent to nnUNet with and without supervision (NDSnnUNet), and SwinUNETR, for a fraction of the trainable parameters (respectively 31.2 and 15.7 Mpms). Figs 4, Fig 5–Fig 6 respectively show the aggregate label Dice score, precision and recall for every fold, and the average ranking of models across folds. Friedman’s *p*-value signals how likely is the result under the hypothesis that models have the same rank distribution across folds. The Critical Difference (CD) diagram shows the average ranking of models across folds, and whether pairwise comparisons survived the conservative Bonferroni-corrected Wilcoxon test, models connected with a line are not significantly different.

### Comparing parameter size and memory with (Swin)UNETR

We implemented SwinUNETR according to the respective paper training settings and MONAI defaults. Regarding SwinUNETR, setting a convolutional feature size entails the same token feature size, which starts at 24, i.e., smaller than Swin-T, the tiny version of 2D SwinTransformers [31]. The SwinTransformer employs a mechanism to reduce the number of tokens, by concatenating neighboring tokens along the feature axis, so it doubles the previous layer feature size. The encoder thus has 4 merge-and-double bottlenecks, and the feature size is 24, 48, 96, 192. Moreover, SwinUNETR was trained on 128×128×128 resolution, affecting the number of positional encodings (relative position biases) required by the SwinTransformer. This SwinUNETR reaches 15 Mpms.

For what concerns the TokenUNet family, the blueprint case is our additive NoTokenUNet, with stages of feature size 16,32,64,128 for a two-fold reason: on the one hand, it is preferable to tokenize a relatively high-resolution feature size, with rich spatial information; on the other hand, the power-of-2 feature size is more common than many UNets’ 320, both for convolutional and Transformer models, making it easier to integrate pretrained models, eventually. It is possible to instantiate a “decoupled” TokenUNet, setting convolutional feature size $d_{c}$ and expanding tokens linearly to a token feature size $d_{t}$ to possibly match several pretrained Transformers or token-mixing architectures of any size. An extending linear map $W_{e}(T):{\mathbb{R}}^{d_{c}}\mapsto {\mathbb{R}}^{d_{t}}$ can be added after TokenLearner, and a shrinking linear projection $W_{s}(T):{\mathbb{R}}^{d_{t}}\mapsto {\mathbb{R}}^{d_{c}}$ added before TokenFuser.

**Fig 7:**
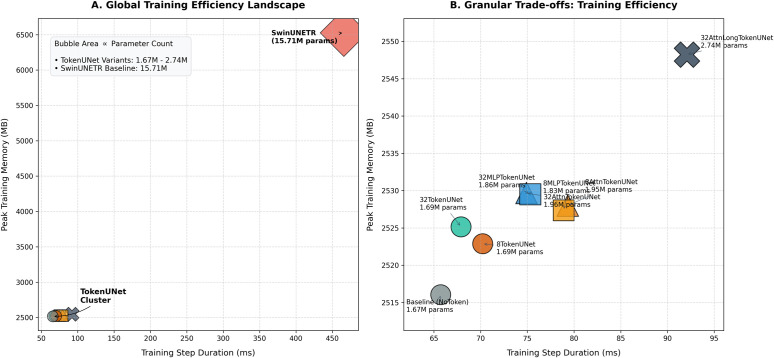
Training memory vs training time occupation on GPU. Bubble size proportional to parameter count.

**Fig 8:**
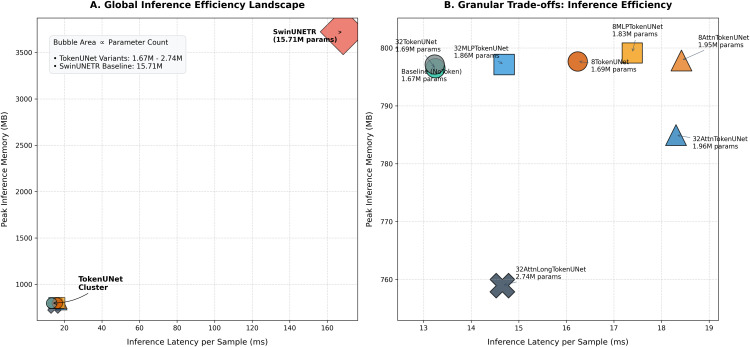
Inference memory vs inference time occupation on GPU. Bubble size proportional to parameter count.

Figs 7 and 8 show how the TokenUNet cluster and SwinUNETR compare respectively in terms of training and inference throughput, memory usage. TokenUNet models are very close to one another, showing how light a Transformer can be when the number of tokens is constrained.

### What TokenLearner learns

**Fig 9:**
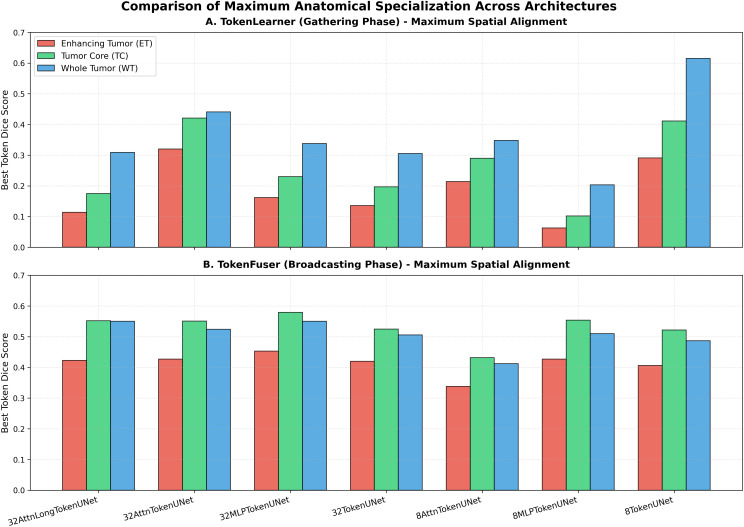
Bar chart of the average in maximal token-label alignment.

**Fig 10:**
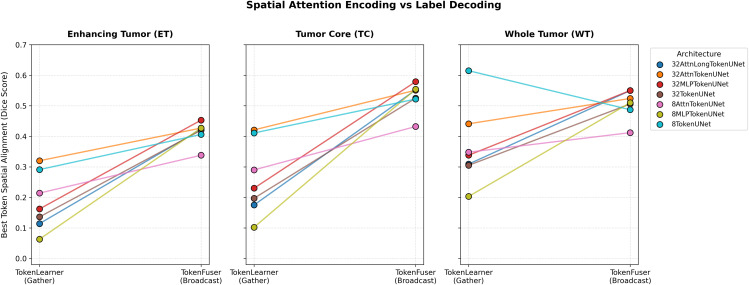
Change in average alignment of the best aligned token between the gathering and broadcasting phase.

TokenLearner gathers spatial information from the feature map and encodes it into token embeddings. TokenFuser broadcasts token embeddings in the same space of feature maps, and in our task the feature maps end in segmentation. We tracked how the spatial attention maps for gathering and broadcasting information aligned with labels, in terms of Dice score. Since every architecture has an independent ordering of tokens in each fold, we only recorded the Dice score of the maximally aligned tokens per label, and averaged it between and across folds. Fig 9 shows for every architecture what was the average Dice score between a label and the maximally aligned token to that label. Fig 10 shows how the maximal alignment changed from TokenLearner spatial attention maps to TokenFuser broadcasting maps.

**Fig 11:**
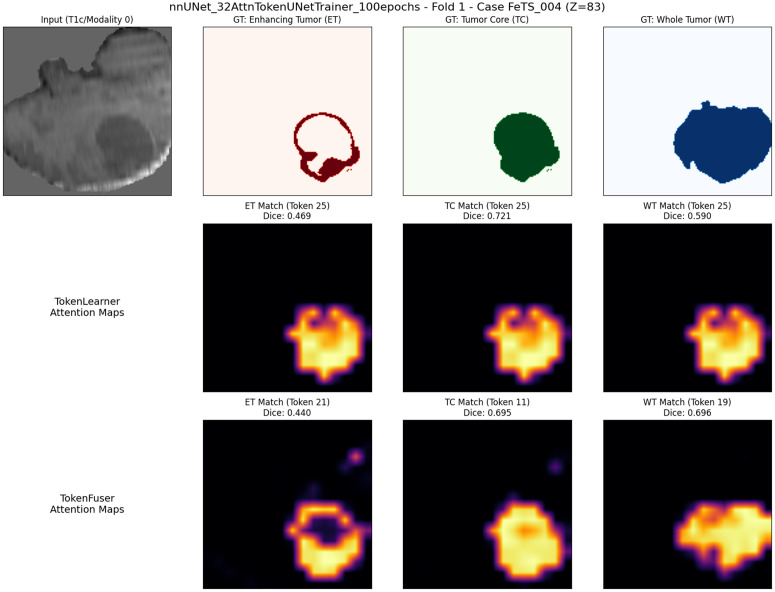
First row shows a random sample T1-weighted scan, and tissue labels. Second row shows TokenLearner’s best aligned token spatial attention maps. Third row shows TokenFuser’s best aligned token spatial broadcast maps.

Fig 11 shows an example of spatial attention and broadcasting maps of a 32AttnTokenUNet, for a random validation sample. This architecture had the highest alignment in both phases respectively, but for the TokenLearner phase the same token was the most aligned with every label, showing little specialization, while for the TokenFuser different tokens aligned with different labels.

## Discussion

Our results generally show how TokenUNets, i.e., an additive UNets with TokenLearner and TokenFuser eventually encasing a sequence or set model (Transformers, MLP-Mixers), match performance of state of art UNets and CNN-Transformer hybrids, with parameters in the order of unit millions instead of tenths of millions, reducing memory footprint and computing time both in training and inference. The performance of TokenUNet demonstrates no significant loss in performance, with effective tokenization. The first explicit aim of TokenLearner is to fix a priori the number of tokens to be processed, thus reducing the computation requirements of downstream blocks, in particular for attention. This brings high flexibility, because a variable number of voxels can be associated to any specific token, and a variable volume size can be tied to a preset number of tokens, whereas ViTs employ fixed associations between the number of pixels (voxels, patches) and tokens, with hindering scaling laws. In those architectures, if the input size is unbound, the number of tokens quickly becomes intractable on most common devices, especially with 3D images. Our models train under a 3GB memory budget on GPU, for batch size of 2. In addition to efficiency, TokenLearner nudges the convolutional encoder layers towards keeping semantically relevant information. This is evidenced by its spatial attention maps, that focus on specific tissues and structure relevant to both domain and task. Experimenting with Transformer-CNN hybrid models with restrained computational resource and maintaining adequate performance satisfies our aim. The attentional maps produced by TokenLearner offer support for analysis of model training modes (either failing or successful) and inference, as well as highlighting features that can guide deep learning practicioners and biomedical scientists to bridge performances and new findings. Compared to post-hoc methods such as Grad-CAM, TokenUNets attention maps are showing the mechanistic process by which the model selects only some regions to compute its output decision Aside from industry and large research centers, and specific competitions, most researchers deal with CPU-only or single-GPU machines that make it increasingly harder to respectively try inference, training, and pretraining of state-of-the-art architectures. Moreover, these adoptions usually require several adaptations to the specific domain and data at hand, further weighting on time and compute resources with attuning loops. By bringing training and deployment on small machines, more research directions could be explored by the community. Development of new models can forgo more iterations given a fixed compute or time budget, allowing statistical testing to choose the most general strategies, avoiding decisions based on random fluctuations, especially in a field characterized by stratified levels of heterogeneity. In parallel, the adoption of innovations and of established architectures of weights can be made easier than before, based on the encasing framework and the code shared with the paper.

### Future directions

Looking forward, the architecture of TokenUNet establishes a pragmatic foundation for several promising research directions, effectively bridging the inductive biases of convolutional layers with the versatility and scaling capabilities of attention mechanisms under strict hardware constraints. A primary direction for future works lies in self-supervised learning (SSL) and pretraining. We hypothesize that semantic tokenization could be more interesting and effective than standard patch-based tokenization for representation learning tasks. By framing the ability to effectively tokenize as a core representation learning objective, self-supervised pretraining on large, unannotated datasets could yield tokens with rich, generalized semantics. This approach has the potential to autonomously uncover previously uncharacterized patterns in the data, or consistently capture subtle pathological features (e.g., myelin scarring in Multiple Sclerosis), that are difficult to explicitly define in strictly supervised regimes, thereby mitigating the chronic label scarcity in biomedical imaging. Furthermore, the TokenLearner module is inherently agnostic to input dimensionality. This structural flexibility opens the door to cross-modal integrations. The highly compressed semantic vectors extracted from 3D medical volumes could seamlessly act as inputs for large-scale Transformers and foundation models natively developed for other modalities, such as clinical text. Exploiting established architectures to process medical imaging tokens could facilitate the development of robust, multi-modal diagnostic tools. Ultimately, by focusing simultaneously on semantic interpretability and strict computational efficiency, the tokenization paradigm of TokenUNet ensures that advanced deep learning modeling remains accessible to the broader scientific community, bounded by common hardware realities rather than elite computing clusters.

### Limitations

It is difficult to validate and measure the true impact of model variants, especially in their clinical side. Every research and clinical task has its own specifics regarding different types of errors. As an example, recall and precision in the glioma usecase could influence the surgery, removing most tumor tissues and least healthy tissue. Our experiments are performed on a single-disease and rare dataset, with multimodal scans for every subject and an already diagnosed condition, apparent in the labels performed by computer-aided experts. While the computational efficiency can be measured and estimated properly, as long as the hardware and software systems are analogous to those of clinical practice (we used 24GB GPUs and proposed models with memory peaks below 3GB in our setting), real world data are inherently more varied than sample datasets, and the effects of model performance cannot be known completely a priori nor they depend single-handedly on the model itself. Future research should start by extending the range of datasets, organ systems, conditions and data modalities on which this segmentation technique is applied. An essential component of model safety in translational research is that of explainable or interpretable results, that allow to understand and even foresee model errors as well as validate correct decisions with experts in the loop. While TokenLearner offers mechanistic interpretability, it is not an explainable AI tool. Mechanistic interpretability comes from the fact that spatial attention maps are not only post hoc visualization tools, such as Grad-CAM heatmaps, but rather the true weight tensors by which the model performs weighted sampling of feature maps. However, the model does not offer an explicit read out as to why a voxel is relevant to a feature and what real world concepts are related to the features, requiring indeed post hoc interpretation to associate channels or tokens to concepts such as “brain,” “whole tumor,” “tumor core,” and so forth. Nonetheless, condensing such primitives into tokens that can be processed by separate models, such as LLMs, offers interesting lines for future research.

## Conclusion

Modern AI has moved towards development of foundation models, i.e., large sized models trained possibly without supervision on immense datasets, and capable to solve new tasks on new data mostly without retraining, or with small scale fine-tuning, or integration with smaller task-specific modules. This thread of development is based mainly on the versatility of the Transformer architecture, its effective combination with modern hardware resources, and consequently on large scale dataset and long lasting training of such models. Biomedical applications of deep learning techniques are often problematic by these terms, because clinical and research computational resources are limited in hardware, bounded in time, and require several domain-dependent adaptations. At the same time, data such as biomedical images are inherently three-dimensional (e.g., MRI, Computed Tomography, etc.) due to the nature of underlying structures of interest, further increasing the computational requirements for processing. Methods able to bring the generality of foundation models to such specialized domains have the potential to accelerate research. In this context, testing the possibility of applying Transformers to different settings is of great importance. Since the block architecture with MultiHead Attention and MLP is not specific to language or vision, recent works have employed Vision Transformers in computer vision architectures, even extending them directly to 3D data. However, task- and domain-specific layers need to be integrated in Transformers, such as linear embeddings from 3D patches to tokens (and back), in the case of 3D biomedical scans. Departing from previous approaches that integrate Transformers in long-time favorite architectures for medical segmentation, we have designed a new frame around the classic ViT. Our models adapt the encoder and decoder components around the Transformer in a novel way, with the explicit aim of reducing memory and computational footprints compared to the direct tokenization of 3D data based on patching. The effect is a great reduction in the resources needed to train and test these models, decoupled from the input resolution, from the number of parameter. In particular, the TokenLearner framework leads to the output of tokens that refer to sparse and interpretable locations of an image, sharing common features. While a token is usually tied to a patch with a specific location, and progressively integrates information from other patch-tokens, with TokenLearner a token may be related to all separate parts of the image containing a specific tissue, or belonging to the same anatomical structure. The process is completely data-driven, and naturally lends to interpretability, which is extremely important in diagnostic-like tasks.

In conclusion, we are offering a new framework for a more efficient addition of Transformers to the set of tools employed in 3D medical imaging, allowing even small labs working with heavy data loads to experiment at the forefront of deep learning technologies.
