# Supplementary material for: TokenUNet: A new case for transformers integration in efficient and interpretable 3D UNets for brain imaging segmentation
Source: PLoS One. 2026 Aug 5;21(8):e0354511. doi: 10.1371/journal.pone.0354511 (PMC13440844; doi:10.1371/journal.pone.0354511)
Supplement: Supplementary file 1 [file pone.0354511.s001.pdf]

Original Manuscript ID: PONE-D-26-10976R1

Original Article Title: “TokenUNet: A New Case for Transformers Integration in Efficient and Interpretable 3D UNets for Brain Imaging Segmentation”

To: Editor and Reviewers

Re: Response to reviewers

Dear Editor,

Thank you very much for the very detailed analysis of the paper and for the extremely useful comments.

We answered each comment by the reviewers and we modified the text according to them.

In response to Reviewers' suggestions, we moved all our experimental analyses to the nnunetv2 framework, applying the very same 5 fold cross-validation protocol to all our proposed and competitor architectures, unifying hyperparameters at every level apart from the architecture peculiarities that define the different models.
This change alone, although computation heavy, solved many of the weak points of the previous version and in our opinion greatly elevates the robustness of our methods and the validity of discussions based on our results.
Importantly, we recorded different metrics and quantified questions regarding the interpretability of spatial attention maps created by TokenLearner and TokenFuser.
We hope our architectures and experimental infrastructure is now both more solid and clear than before the first round.
We deeply thank our reviewers for motivating these changes.

We are uploading (a) our point-by-point response to the reviewers’ comments (below), (b) an updated manuscript with yellow highlighting indicating changes, and (c) a clean updated manuscript without highlights.

Best regards,

The Authors

Introductory response for Reviewer 1

Dear Reviewer 1,

Thank you for your commitment to our manuscript.
In the following you will find a response to the points you raised.

Best regards,
The Authors

Reviewer#1, Concern #1

"For the number of Tokens (N), the author only adopted a fixed value of N=8, lacking specific selection basis and failing to conduct parameter sensitivity analysis. It is necessary to supplement comparative experiments with different token numbers such as N=4, 8, 16, and 32 to illustrate the basis for the optimal setting and stability"

> **Author response:** It is true that we adopted a fixed value, which we wanted to be minimal for computational reasons, and larger than the number of output labels for flexibility. The number of tokens N=8 is the minimal setting proposed in the original TokenLearner work, which also uses N=16. We agree that it is worthwhile to expand the experiments with different token numbers.
>
> **Author action:** As a trade-off between exploration of different numbers, and exploitation of efficient settings, we decided to only experiment with N=8 and N=32.

Reviewer#1, Concern #2

"In Section 3.3, the author does not specify the data augmentation strategies, augmentation types, and corresponding parameters used during training."

> **Author response:** The reviewer is correct in noticing the missing specification. We agree that the augmentation settings are a key part of training and evaluation with regard to generalizations. We have now reframed our experiments into the nnUNet methodology, and we are detailing the augmentations performed in its recipe.
>
> **Author action:** We have complemented the Method Section with descriptions of the spatial, noise and intensity data augmentations native to the nnunet framework, from line 278.

Reviewer#1, Concern #3

"The paper exhibits inconsistent tense usage when describing specific completed research activities, including model training, data processing, and result analysis. For concrete experimental operations, data processing procedures, model training setups, and the research results obtained in this study, the simple past tense should be used consistently."

> **Author response:** Thank you for highlighting this problem that may hinder readability of the paper.
>
> **Author action:** We have harmonized the past tense of the manuscript for concrete procedures and results, specifically in the Method and Results Sections.

Introductory response for Reviewer 2

Dear Reviewer 2,

Thank you for your commitment to our manuscript.
In the following you will find a response to the points you raised.

Best regards,
The Authors

Reviewer#2, Concern #1

"While the use of TokenLearner is interesting, the manuscript does not sufficiently clarify how this contribution advances beyond existing token-reduction or efficient attention mechanisms. Moreover, recent developments in efficient Transformers, lightweight attention, and hybrid CNN-Transformer architectures are not thoroughly discussed. A stronger and more critical comparison with recent state-of-the-art approaches is necessary to convincingly establish the novelty and significance of the proposed method."

> **Author response:** Thank you for highlighting missing connections to extensive development in deep learning architectures. We have focused on readily available, torch-first, first principle approaches, compared for example to low-level development such as new CUDA kernels and hardware-friendly implementations of mathematical operations; on the architecture side, we have considered the developments that consistently occur in the medical imaging field, namely in the BraTS challenge, where for example the Swin-UNETR is a renowned hybrid CNN-Transformer architecture. However these different lenses were not extensively compared and discussed in the first version of our manuscript.
>
> **Author action:** We extended the contextualization of efficiency-based approaches and developments related to our architectural approach in the Related Works subsection of the Introduction (from line 126 in particular).

Reviewer#2, Concerns #2, #4

"The experimental evaluation lacks sufficient rigor and breadth. Although the authors compare their method with UNet and SwinUNETR, the evaluation is limited to a single dataset (FeTS/BraTS subset) and a single metric (Dice score) . This is insufficient to demonstrate generalizability. Additional evaluation on other datasets or tasks would significantly strengthen the claims. Furthermore, no statistical significance testing is provided to support the reported performance differences (e.g., 87.21% vs 86.75% Dice), which are

relatively small. Without statistical validation, it is unclear whether these improvements are meaningful or within variance."

"The manuscript relies solely on Dice score for evaluation. While Dice is a standard metric for segmentation, it does not fully capture model performance in clinical settings. Metrics such as Hausdorff distance, sensitivity, specificity, or region-wise performance would provide a more comprehensive evaluation. Additionally, no discussion is provided regarding clinical significance or potential deployment implications, which is important given the biomedical application domain."

> **Author response:** Thank you for underlining the importance of statistical testing and multiple evaluation measures. We failed to express how we do not aim to achieve superiority on a single measures; rather we aimed to approximate parity within standard errors under reduced computation, which we managed. Nonetheless, we moved to full compatibility with the nnunetv2 protocol and now embrace the use of multiple performance measures, and follow the FeTS/BraTS example by reporting not only Dice score but also precision and recall, in order to highlight the models' tendencies towards false positive and false negative segmentations. While the images cover aggregate measures, the code will open source and show all per-label results, adding IoU, TP, TN, FP, FN. Moreover, we have discussed the implications of these aspects in translational, clinical domains.
>
> Finally, we agree that using more datasets would strengthen the claims of generalizability, however there is a lack of suitable multimodal datasets for brain segmentation, and thus we leave the extension of such experiments to future research, while discussing explicitly their importance.
>
> **Author action:** We expanded the set of performance measures, highlighted where performance differences are higher than sample variance, and discussed the implications in Results Fig 5 and 6 in particular. We have expanded on translational limits in the Limitations subsection in the Discussion Section, from line 417.

Reviewer#2, Concern #3

"A critical issue is the lack of fair comparison across models. The manuscript explicitly states that hyperparameter tuning was avoided to reduce computational cost. While this aligns with the goal of efficiency, it raises concerns about whether competing models (especially SwinUNETR) were optimally configured. Differences in optimization strategies (SGD vs AdamW, different learning rates) further complicate fair comparison . This may bias the results in favor of the proposed model. A fair comparison requires consistent tuning protocols or justification that all models are equally optimized."

> **Author response:** Thank you for raising the point. While we tried to use the parameters suggested in the papers presenting each model class, it is true that the set up makes it hard to evaluate whether the comparison in itself is fair, because the degrees of freedom are growing exponentially. For this reason we have revised our experimental battery in order to use the nnUNet suggested defaults for all model classes, thus evaluating fairly which models are the best for these widely accepted settings.
>
> **Author action:** We moved our evaluations to a battery of models implemented in nnUNet cross validations with default identical hyperparameters.

Reviewer#2, Concern #5

"Although the authors mention that code will be released upon publication, reproducibility remains limited in the current submission. Important details such as hyperparameter sensitivity, initialization variability, and robustness across different training conditions are not discussed. Moreover, the use of batch size 1 with

gradient accumulation and specific architectural simplifications may impact reproducibility across different hardware environments.”

> **Author response:** We agree that the first submission, as noted in other reviewers’ concern, was missing key information to reproduce unambiguously our results. We have now both enhanced the clarity of descriptions in our pipeline, as well as published a repository to actually inspect and run the analyses coming with the reviewed manuscript. Regarding the use of gradient accumulation and batch size=1, we avoid all related problems by adopting the nnUNet framework..
>
> **Author action:** We are publishing code for the revision, we described augmentations and performance measures in the manuscript, and highlighted the potential differences of running such experiments on different GPUs in the Limitation subsection.

Reviewer#2, Concern #6

“The manuscript occasionally overstates its contributions. For example, the claim that the model “tops” SwinUNETR performance is based on a relatively small improvement in Dice score, which may not be statistically significant. Similarly, claims regarding interpretability through attention maps are not quantitatively evaluated. While visualizations (e.g., attention maps in Figure 7) are useful, they remain qualitative and require more rigorous validation.”

> **Author response:** The reviewer is correct in that the model does not “top” SwinUNETR in pure performance. The correct statement is that our model on average reached SwinUNETR’s performance, for a fraction of the cost, topping it in terms of efficiency. Regarding interpretability, while explainable and interpretable AI are subdomains of their own, with proper quantification of what is meant by “explainable” and “interpretable”, our models are only qualitatively interpretable, but inherently so, with the important addition that attention maps are a priori mechanistically responsible for the computations and decisions of our model, because they dictate which voxels are sampled and at what strength, whereas attention maps based on gradients such as Grad-CAM are post-hoc interpretation tools.
>
> **Author action:** We have elucidated the differences between interpretability of TokenLearner attention maps, Grad-CAM-like methods, with pointers to the domains of explainability and interpretability research in Related Works as well as Discussion Section, Limitation subsection (lines 150 and 435).

Introductory response for Reviewer 3

Dear Reviewer 3,

Thank you for your commitment to our manuscript.
In the following you will find a response to the points you raised.

Best regards,
The Authors

Reviewer#3, Concern #1

“The manuscript positions Token-UNet primarily as a new and efficient case for Transformer integration. However, the ablation narrative indicates that the largest performance gain is obtained from the transition from the classic UNet baseline to the modified UNet** backbone, while the Transformer itself appears to

contribute less than the TokenLearner and TokenFuser bottleneck. This is a crucial point because it affects how the novelty should be framed. The authors should revise the title, abstract, introduction, and conclusion so that the stated contribution is fully aligned with the experimental evidence. It should be made explicit whether the main innovation is the token bottleneck design, the revised additive UNet backbone, the efficient insertion of a lightweight Transformer, or the combined architectural pipeline."

Author response: The reviewer is right in addressing the possible confusion in our contributions and their relevance to the field.
First of all, we clarify that our aim is not that of achieving state-of-art results in performance metrics.
Our contributions should be framed as such, building the literal "case" that can box Transformer-like models in an efficient way, and pushing forward the case for doing so:
1) the UNet backbone is reinforced in order to comply with the goals of small memory and time complexity/occupation, modularity, and the original aim of UNets in general;
2) the token bottleneck is a stand-alone means to implement an effective information bottleneck with interpretable characteristics, as well as an established tokenization alternative that we suggest should be adopted widespread when considering hybrid CNN-Transformer architectures.
The addition of a basic Transformer is a proof of concept that the tokenization scheme is effective and makes such Transformers more efficient than standard tokenization by voxel patches.
We leave to future works to fully demonstrate the advantage of pretraining and fine-tuning large Transformers, since in other domains it has had the largest returns.
Meanwhile we highlight a strong case for doing so efficiently.

Author action: We have better framed the Introduction of our main contribution, as well as the discussion of our future objectives and suggestions for the research community to expand on these results, in the Future directions subsection of our Discussions.

Reviewer#3, Concern #2, #3, #4, #5

"Insufficient baseline breadth for a strong segmentation claim.
Fairness of the optimisation and training protocol.
Evaluation metrics are too narrow for a medical segmentation study.
Statistical analysis should be strengthened."

Author response: We agree with the reviewer that the best generalist baseline is the nnU-Net framework. We have thus decided to rerun our pipeline with both its baseline 3D architecture, as well as our previous comparative baselines.

Author action: We completely renovated both the Methods and the Result section in order to implement the reviewer suggestion and make a strong case for our recalibrated claims. We evaluate 5-fold cross validations of several models using the same hyperparameters and training recipe, identifying a variant that performs statistically better, which is a target for further developments with personalized hyperparameters.

Reviewer#3, Concern #6

"All experiments are conducted on a single dataset under internal five-fold cross-validation. While this is a reasonable starting point, the manuscript occasionally generalises its conclusions to 3D biomedical imaging more broadly. At present, the evidence supports only a more limited conclusion tied to this specific tumour segmentation setting. The discussion and conclusion should therefore be moderated unless additional validation is provided. Ideally, the revised manuscript should include an external validation experiment, a

second dataset, or at minimum a more explicit acknowledgement of the limits of the current evidence base."

Author response: We agree that our results and thus our claims are confined to the specific setting of the 1 task of segmentation for the 1 dataset of BraTS/FeTS. While this dataset presents a wide array of scanners, settings, MRI modalities and subjects, across multiple centers and many years, it is not enough to generalise conclusions more widely. Our scope is indeed limited at the moment.

Author action: We are specifically addressing the issue in the Limitations and Future Work sections of the revised manuscript.

Reviewer#3, Concern #7

"The manuscript indicates that the code will be released upon publication, but it currently provides only a placeholder rather than a concrete repository. In addition, several implementation details remain insufficiently specified, including preprocessing steps, normalisation strategy, fold generation control, augmentation pipeline, model selection protocol, and precise inference configuration. Since the manuscript emphasises efficiency and accessibility, reproducibility should be one of its strongest features. The revised version should provide a substantially clearer experimental protocol and, if possible, an accessible code repository for review."

Author response: The code is now available as a repository containing the custom folders added to the official nnunetv2 repository, and the added scripts and notebooks to reproduce our experiments.

Author action: We link our added repository in the paper.

Reviewer#3, Concern #8

"A notable strength of the paper is the attempt to provide interpretable token attention maps. However, the interpretability analysis remains largely qualitative and appears to rely on a small number of visual examples. This is not yet sufficient to support a strong interpretability claim. The authors should provide a more systematic evaluation of the attention maps, for example by analysing multiple cases, quantifying correspondence with lesion regions, or including expert assessment of whether the highlighted regions are stable and clinically meaningful. As it stands, the interpretability discussion is promising but preliminary."

Author response: We agree with the reviewer's concern that our first illustrations were only preliminary. Thus we have decided to move towards a succinct mathematical analysis of alignment between TokenLearner and TokenFuser generated maps, with the ground truth tumor labels. This is accompanied by examples (expanded on in the companion repository) that show attention maps and labels in an hopefully clearer and informative way.

Author action: We have systematically analyzed token map and label alignment in terms of Dice score, across all models and folds, and visualized these data and an example of labels and attention activations in our Result Section, with Figures 9, 10, 11.

Reviewer#3, Concern #9

"Several important architectural decisions appear to be fixed without adequate justification, most notably the number of learned tokens and the depth or width of the Transformer component. Because the central premise of the work is an efficiency-performance trade-off, these parameters should not remain unexplored. The revised manuscript would benefit substantially from a sensitivity study showing how token

count and Transformer complexity affect memory usage, inference time, and segmentation quality. This would help establish whether the chosen configuration is principled or merely one reasonable setting among many."

Author response: We agree that, although computationally burdensome, an experimental comparison along some of these architectural parameters dimensions would be essential. We have decided to report the computational trade-offs in inference and training time, memory, for a battery of models that span 8, 32 tokens, either directly fed from encoder to decoder or passed through a 2-blocks Transformer, a 2-blocks MLP-Mixer, and at the extreme of 32 tokens also an 8 blocks Transformer. In this range, all models perform in a remarkably similar manner, even in terms of computational requirements.

Author action: We have expanded our results with an efficiency landscape exploration exemplified in Figures 7 and 8.

Reviewer#3, Concern #10

"The paper repeatedly emphasises operation on common or constrained hardware, yet the experiments were conducted on a workstation-class GPU with 24 GB memory. Although the relative efficiency improvements are important, the phrase common hardware may overstate the practical accessibility demonstrated by the current study. The authors should either moderate this language or provide additional evidence showing that the proposed framework remains viable under more modest hardware conditions, such as lower-memory GPUs or reduced deployment settings."

Author response: We acknowledge the fact that the specified hardware we have worked on is fairly performant, although many winning submissions to the BraTS and FeTS challenges have used even more performant hardware. However, the original analysis was performed also for CPU (again, workstation level), and regarding the GPU memory usage, we have written out the actual usage which is below the full limit of our hardware. This is a useful indication, since a model occupying around 3GB of memory on a 24GB hardware will occupy cca 3GB of memory also on 8GB hardware, as long as both set-ups can run compatible CUDA and PyTorch versions, even if at different speed, and we believe that this makes a sensible difference in the democratization of models, even when hardware and computation time are rented. We thus think we are giving useful information for "safe bets" for researchers deciding on which models to implement and further modify.

Author action: We have clarified in the Limitations section how our hardware may not be commonly available and how translation to more modest set ups may not be 1:1, even if more promising than high-compute competitor architectures.

Reviewer#3, Concern #11

"The manuscript concludes with broad claims regarding the democratisation of foundation-model style methods for biomedical imaging. While this is an interesting future direction, the present experimental study does not yet establish such a claim. The discussion should more carefully distinguish between demonstrated findings, plausible interpretations, and future possibilities. A more disciplined conclusion would increase the credibility of the manuscript."

Author response: We agree that the lack of a clearly separate Limitation subsection had shaded the differences between demonstrated findings and future possibilities.

Author action: We have separated our discussions with Limitations and Future Works subsections and made clearer distinctions between demonstrated facts, plausible hypotheses and desired developments.

Reviewer#3, Concern #12

"The manuscript contains several wording issues, typographical errors, and minor presentation inconsistencies that reduce readability. In addition, some figure captions and explanatory statements would benefit from clearer and more self-contained phrasing. A careful language revision is recommended to improve precision, professionalism, and overall readability."

> **Author response:** We share the desire for the best readability of our work and the need for best effort revisions. We thank the reviewers for bringing up the issue.
>
> **Author action:** We have checked and rewritten paragraphs and captions, hopefully elevating readability..

Introductory response for Reviewer 4

Dear Reviewer 4,

Thank you for your commitment to our manuscript.
In the following you will find a response to the points you raised.

Best regards,
The Authors

Reviewer#4, Concern #1

"Add the names of the datasets used in the abstract."

> **Author response:** Thanks for highlighting the lack of such grounding information in the access point of our manuscript.
>
> **Author action:** We have made clear in the abstract that we are working on BraTS challenge data, for the task of glioma segmentation.

Reviewer#4, Concern #2

"Explaining research contributions in clear and simple bullet points."

> **Author response:** We agree that bullet points are helpful for summarizing contributions.
>
> **Author action:** We have added a bullet point list in the Introduction Section.

Reviewer#4, Concern #3

"Add a table summarizing the related work in terms of the name of the dataset used, the techniques and metrics adopted, as well as the advantages and disadvantages of each research paper."

> **Author response:** We agree with the reviewer that tables are another essential summarizing tool. However, since our research somehow side-steps common objectives in the domain and is not concerned primarily with performance metrics, nor extended to other datasets yet, we have

decided to restructure a Related Works section that recovers the related architectures, as well as different approaches to reduce attention computations, and different approaches to interpretability of model decisions

Author action: We have restructured the Related Works subsection.

Reviewer#4, Concern #4

“Summarize the conclusions in a way that reflects only the most important findings of the researcher and proposed future work.”

Author response: We recognize that our Conclusion Section was partly redundant with a discussion section. We have cut paragraphs and restructured also the Discussion Section itself, so that now we have Future directions and Limitation as subsections of Discussion, and an edited Conclusion that we hope is a lighter and clearer wrap-up of the manuscript.

Author action: We have jointly reworked the Discussion and Conclusion sections.

Reviewer#4, Concern #5

“Add references for the period between 2025-2026, with no less than two references for each year.”

Author response: We agree that focusing on new architectures for the specific BraTS challenge gave a distorted view of the more modern literature, because of natural publication timelines as well as the competitive aspect of some innovations. However we have added

Author action: We have added references such as Khan et al. (IEEE Access. 2025), Wu B, Yang C. ((NNICE). IEEE; 2025), Parida A et al. (Springer; 2025.), Sabanci et al. (Bioengineering; 2026), Xu Y, et al. (Quantitative Imaging in Medicine and Surgery. 2026)
