# Supplementary material for: TokenUNet: A new case for transformers integration in efficient and interpretable 3D UNets for brain imaging segmentation
Source: PLoS One. 2026 Aug 5;21(8):e0354511. doi: 10.1371/journal.pone.0354511 (PMC13440844; doi:10.1371/journal.pone.0354511)
Supplement: Peer review history

# Author comment: Author response to Decision Letter 0

**DOI:** 10.1371/journal.pone.0354511.r001

**Transfer Alert**

This paper was transferred from another journal. As a result, its full editorial history (including decision letters, peer reviews and author responses) may not be present.

4 Mar 2026

# Peer review: Decision Letter 0

**DOI:** 10.1371/journal.pone.0354511.r002

**Authors:** Kumaradevan Punithakumar

29 Apr 2026

PONE-D-26-10976

Token-UNet: A New Case for Transformers Integration in Efficient and Interpretable 3D UNets for Brain Imaging Segmentation

PLOS One

Dear Dr. Tshimanga,

Thank you for submitting your manuscript to PLOS ONE. After careful consideration, we feel that it has merit but does not fully meet PLOS ONE’s publication criteria as it currently stands. Therefore, we invite you to submit a revised version of the manuscript that addresses the points raised during the review process.

Please submit your revised manuscript by Jun 13 2026 11:59PM. If you will need more time than this to complete your revisions, please reply to this message or contact the journal office at plosone@plos.org. When you're ready to submit your revision, log on to https://www.editorialmanager.com/pone/ and select the 'Submissions Needing Revision' folder to locate your manuscript file.

Please include the following items when submitting your revised manuscript:

- A letter that responds to each point raised by the academic editor and reviewer(s). You should upload this letter as a separate file labeled 'Response to Reviewers'.
- A marked-up copy of your manuscript that highlights changes made to the original version. You should upload this as a separate file labeled 'Revised Manuscript with Track Changes'.
- An unmarked version of your revised paper without tracked changes. You should upload this as a separate file labeled 'Manuscript'.

If you would like to make changes to your financial disclosure, please include your updated statement in your cover letter. Guidelines for resubmitting your figure files are available below the reviewer comments at the end of this letter.

If applicable, we recommend that you deposit your laboratory protocols in protocols.io to enhance the reproducibility of your results. Protocols.io assigns your protocol its own identifier (DOI) so that it can be cited independently in the future. For instructions see: https://journals.plos.org/plosone/s/submission-guidelines#loc-laboratory-protocols. Additionally, PLOS ONE offers an option for publishing peer-reviewed Lab Protocol articles, which describe protocols hosted on protocols.io. Read more information on sharing protocols at https://plos.org/protocols?utm_medium=editorial-email&utm_source=authorletters&utm_campaign=protocols.

As the corresponding author, your ORCID iD is verified in the submission system and will appear in the published article. PLOS supports the use of ORCID, and we encourage all coauthors to register for an ORCID iD and use it as well. Please encourage your coauthors to verify their ORCID iD within the submission system before final acceptance, as unverified ORCID iDs will not appear in the published article. *Only*  the individual author can complete the verification step; PLOS staff *cannot*  verify ORCID iDs on behalf of authors.

We look forward to receiving your revised manuscript.

Kind regards,

Kumaradevan Punithakumar

Academic Editor

PLOS One

Journal Requirements:

When submitting your revision, we need you to address these additional requirements.

1. Please ensure that your manuscript meets PLOS ONE's style requirements, including those for file naming. The PLOS ONE style templates can be found at

https://journals.plos.org/plosone/s/file?id=wjVg/PLOSOne_formatting_sample_main_body.pdf and

https://journals.plos.org/plosone/s/file?id=ba62/PLOSOne_formatting_sample_title_authors_affiliations.pdf

2. Please note that PLOS One has specific guidelines on code sharing for submissions in which author-generated code underpins the findings in the manuscript. In these cases, we expect all author-generated code to be made available without restrictions upon publication of the work. Please review our guidelines at https://journals.plos.org/plosone/s/materials-and-software-sharing#loc-sharing-code and ensure that your code is shared in a way that follows best practice and facilitates reproducibility and reuse.

3. Please update your submission to use the PLOS LaTeX template. The template and more information on our requirements for LaTeX submissions can be found at http://journals.plos.org/plosone/s/latex.

4. Thank you for stating the following financial disclosure:

“This work was supported by the STARS@UNIPD funding program of the University

of Padova, Italy, through the project: MEDMAX, https://www.unipd.it/stars.

This project has received funding from the

European Union’s Horizon Europe research and innovation programme under grant agreement no

101137074 - HEREDITARY https://hereditary-project.eu/ https://commission.europa.eu/funding-tenders/find-funding/eu-funding-programmes/horizon-europe_en”

Please state what role the funders took in the study. If the funders had no role, please state: "The funders had no role in study design, data collection and analysis, decision to publish, or preparation of the manuscript."

If this statement is not correct you must amend it as needed.

Please include this amended Role of Funder statement in your cover letter; we will change the online submission form on your behalf.

5. Thank you for stating the following in the Acknowledgments Section of your manuscript:

“This work was supported by the STARS@UNIPD funding program of the University of Padova, Italy, through the project: MEDMAX. This project has received funding from the European Union’s Horizon Europe research and innovation programme under grant agreement no 101137074- HEREDITARY.”

We note that you have provided funding information that is not currently declared in your Funding Statement. However, funding information should not appear in the Acknowledgments section or other areas of your manuscript. We will only publish funding information present in the Funding Statement section of the online submission form.

Please remove any funding-related text from the manuscript and let us know how you would like to update your Funding Statement. Currently, your Funding Statement reads as follows:

“This work was supported by the STARS@UNIPD funding program of the University

of Padova, Italy, through the project: MEDMAX, https://www.unipd.it/stars.

This project has received funding from the

European Union’s Horizon Europe research and innovation programme under grant agreement no

101137074 - HEREDITARY https://hereditary-project.eu/ https://commission.europa.eu/funding-tenders/find-funding/eu-funding-programmes/horizon-europe_en”

Please include your amended statements within your cover letter; we will change the online submission form on your behalf.

6. We note you have included a table to which you do not refer in the text of your manuscript. Please ensure that you refer to Table 2 in your text; if accepted, production will need this reference to link the reader to the Table.

7. If the reviewer comments include a recommendation to cite specific previously published works, please review and evaluate these publications to determine whether they are relevant and should be cited. There is no requirement to cite these works unless the editor has indicated otherwise.

[Note: HTML markup is below. Please do not edit.]

Reviewers' comments:

Reviewer's Responses to Questions

**Comments to the Author**

1. Is the manuscript technically sound, and do the data support the conclusions?

The manuscript must describe a technically sound piece of scientific research with data that supports the conclusions. Experiments must have been conducted rigorously, with appropriate controls, replication, and sample sizes. The conclusions must be drawn appropriately based on the data presented.

Reviewer #1: Yes

Reviewer #2: Partly

Reviewer #3: Partly

Reviewer #4: Yes

**********

2. Has the statistical analysis been performed appropriately and rigorously?

Reviewer #1: Yes

Reviewer #2: Yes

Reviewer #3: N/A

Reviewer #4: Yes

**********

3. Have the authors made all data underlying the findings in their manuscript fully available?

The PLOS Data policy requires authors to make all data underlying the findings described in their manuscript fully available without restriction, with rare exception (please refer to the Data Availability Statement in the manuscript PDF file). The data should be provided as part of the manuscript or its supporting information, or deposited to a public repository. For example, in addition to summary statistics, the data points behind means, medians and variance measures should be available. If there are restrictions on publicly sharing data—e.g. participant privacy or use of data from a third party—those must be specified.

Reviewer #1: Yes

Reviewer #2: Yes

Reviewer #3: No

Reviewer #4: Yes

**********

4. Is the manuscript presented in an intelligible fashion and written in standard English?

PLOS ONE does not copyedit accepted manuscripts, so the language in submitted articles must be clear, correct, and unambiguous. Any typographical or grammatical errors should be corrected at revision, so please note any specific errors here.

Reviewer #1: Yes

Reviewer #2: Yes

Reviewer #3: Yes

Reviewer #4: Yes

**********

5. Review Comments to the Author

Please use the space provided to explain your answers to the questions above. You may also include additional comments for the author, including concerns about dual publication, research ethics, or publication ethics. (Please upload your review as an attachment if it exceeds 20,000 characters)

Reviewer #1: To address key issues in Transformer-based 3D medical image segmentation, such as high computational complexity, strong hardware dependence, and difficulties in clinical deployment, the author proposes Token-UNet, a lightweight hybrid architecture. It achieves adaptive semantic tokenization via the TokenLearner and TokenFuser modules, efficiently integrating a lightweight Transformer into the 3D UNet framework. A series of validation experiments demonstrate that the model achieves simultaneous improvements in accuracy and efficiency for brain tumor segmentation, while also exhibiting favorable interpretability.

Main Deficiencies and Suggestions for Revision�

1.For the number of Tokens (N), the author only adopted a fixed value of N=8, lacking specific selection basis and failing to conduct parameter sensitivity analysis. It is necessary to supplement comparative experiments with different token numbers such as N=4, 8, 16, and 32 to illustrate the basis for the optimal setting and stability.

2.In Section 3.3, the author does not specify the data augmentation strategies, augmentation types, and corresponding parameters used during training. Data augmentation is a critical training component in 3D medical image segmentation that affects model generalization, robustness, and final performance. It is recommended to supplement full details of all data augmentation methods employed and the key parameters for each augmentation technique.

3.The paper exhibits inconsistent tense usage when describing specific completed research activities, including model training, data processing, and result analysis. For concrete experimental operations, data processing procedures, model training setups, and the research results obtained in this study, the simple past tense should be used consistently.

Reviewer #2: 1. Novelty and Positioning with Respect to Literature:

The manuscript claims novelty in integrating TokenLearner and TokenFuser within a UNet framework to reduce computational complexity. However, the idea of combining convolutional encoders with Transformer modules is already well established (e.g., TransUNet, UNETR, SwinUNETR). While the use of TokenLearner is interesting, the manuscript does not sufficiently clarify how this contribution advances beyond existing token-reduction or efficient attention mechanisms. Moreover, recent developments in efficient Transformers, lightweight attention, and hybrid CNN-Transformer architectures are not thoroughly discussed. A stronger and more critical comparison with recent state-of-the-art approaches is necessary to convincingly establish the novelty and significance of the proposed method.

2. Experimental Design and Evaluation Limitations:

The experimental evaluation lacks sufficient rigor and breadth. Although the authors compare their method with UNet and SwinUNETR, the evaluation is limited to a single dataset (FeTS/BraTS subset) and a single metric (Dice score) . This is insufficient to demonstrate generalizability. Additional evaluation on other datasets or tasks would significantly strengthen the claims. Furthermore, no statistical significance testing is provided to support the reported performance differences (e.g., 87.21% vs 86.75% Dice), which are relatively small. Without statistical validation, it is unclear whether these improvements are meaningful or within variance.

3. Lack of Fair Comparison and Hyperparameter Tuning Strategy:

A critical issue is the lack of fair comparison across models. The manuscript explicitly states that hyperparameter tuning was avoided to reduce computational cost . While this aligns with the goal of efficiency, it raises concerns about whether competing models (especially SwinUNETR) were optimally configured. Differences in optimization strategies (SGD vs AdamW, different learning rates) further complicate fair comparison . This may bias the results in favor of the proposed model. A fair comparison requires consistent tuning protocols or justification that all models are equally optimized.

4. Limited Evaluation Metrics and Clinical Relevance:

The manuscript relies solely on Dice score for evaluation. While Dice is a standard metric for segmentation, it does not fully capture model performance in clinical settings. Metrics such as Hausdorff distance, sensitivity, specificity, or region-wise performance would provide a more comprehensive evaluation. Additionally, no discussion is provided regarding clinical significance or potential deployment implications, which is important given the biomedical application domain.

5. Reproducibility and Transparency:

Although the authors mention that code will be released upon publication, reproducibility remains limited in the current submission. Important details such as hyperparameter sensitivity, initialization variability, and robustness across different training conditions are not discussed. Moreover, the use of batch size 1 with gradient accumulation and specific architectural simplifications may impact reproducibility across different hardware environments.

6. Interpretation and Claims:

The manuscript occasionally overstates its contributions. For example, the claim that the model “tops” SwinUNETR performance is based on a relatively small improvement in Dice score, which may not be statistically significant. Similarly, claims regarding interpretability through attention maps are not quantitatively evaluated. While visualizations (e.g., attention maps in Figure 7) are useful, they remain qualitative and require more rigorous validation.

Reviewer #3: Overall recommendation Major Revision

General assessment

This manuscript addresses an important and timely problem in 3D medical image segmentation, namely the high computational burden associated with Transformer-based architectures. The proposed Token-UNet framework is potentially valuable because it attempts to preserve global contextual modelling while substantially reducing memory consumption, inference time, and parameter count. The manuscript is also strengthened by the effort to incorporate qualitative interpretability through TokenLearner attention maps. However, although the study is promising, the current version does not yet provide sufficiently rigorous experimental validation, methodological clarification, or evidence breadth to support several of its stronger claims. In particular, the present framing of the contribution, the fairness of baseline comparison, the scope of evaluation metrics, and the strength of the interpretability analysis require substantial revision before the work can be considered for publication.

Major comments

1. Clarification of the real technical contribution

The manuscript positions Token-UNet primarily as a new and efficient case for Transformer integration. However, the ablation narrative indicates that the largest performance gain is obtained from the transition from the classic UNet baseline to the modified UNet** backbone, while the Transformer itself appears to contribute less than the TokenLearner and TokenFuser bottleneck. This is a crucial point because it affects how the novelty should be framed. The authors should revise the title, abstract, introduction, and conclusion so that the stated contribution is fully aligned with the experimental evidence. It should be made explicit whether the main innovation is the token bottleneck design, the revised additive UNet backbone, the efficient insertion of a lightweight Transformer, or the combined architectural pipeline.

2. Insufficient baseline breadth for a strong segmentation claim

The current experimental comparison is too limited for a study in 3D brain tumour segmentation. A comparison against vanilla UNet and SwinUNETR is informative, but not sufficient to support broad claims about performance competitiveness or architectural superiority in modern medical image segmentation. The omission of nnU-Net is particularly important, given its relevance as a strong and widely accepted baseline in this domain. The study should be expanded to include stronger and more representative baselines, especially methods that are recognised for robust performance under rigorous validation settings.

3. Fairness of the optimisation and training protocol

The proposed models and the SwinUNETR baseline are trained with different optimisers and different learning rates. At the same time, the manuscript states that hyperparameter tuning was intentionally avoided. This creates a concern regarding experimental fairness, because the observed differences may partly arise from optimisation settings rather than architecture alone. The authors should either justify the exact training configuration as faithful reproductions of standard implementations from the literature or provide a controlled comparison under harmonised optimisation conditions. Without this clarification, the relative performance claims remain difficult to interpret with confidence.

4. Evaluation metrics are too narrow for a medical segmentation study

The manuscript reports final segmentation performance only in terms of Dice score. This is not sufficient for a medical image segmentation article, particularly for brain tumour sub-region analysis. Performance should be reported separately for whole tumour, tumour core, and active tumour, and additional metrics such as Hausdorff distance, sensitivity, precision, and possibly specificity should be included. Dice alone does not adequately capture boundary quality, small lesion behaviour, or false positive versus false negative trade-offs. The inclusion of a more complete metric set is necessary to assess whether the proposed efficiency gains are achieved without clinically relevant degradation.

5. Statistical analysis should be strengthened

The manuscript uses descriptive comparisons and boxplots, but it does not provide sufficiently formal statistical testing to support claims of superiority or equivalence. If performance differences are to be interpreted meaningfully, the authors should report appropriate statistical tests across folds, together with confidence intervals and effect sizes where relevant. This is especially important because some reported performance differences are relatively small. Statements such as reaching, topping, or surpassing competing methods should be used only when supported by clear statistical evidence.

6. Generalisability is not yet demonstrated

All experiments are conducted on a single dataset under internal five-fold cross-validation. While this is a reasonable starting point, the manuscript occasionally generalises its conclusions to 3D biomedical imaging more broadly. At present, the evidence supports only a more limited conclusion tied to this specific tumour segmentation setting. The discussion and conclusion should therefore be moderated unless additional validation is provided. Ideally, the revised manuscript should include an external validation experiment, a second dataset, or at minimum a more explicit acknowledgement of the limits of the current evidence base.

7. Reproducibility remains incomplete

The manuscript indicates that the code will be released upon publication, but it currently provides only a placeholder rather than a concrete repository. In addition, several implementation details remain insufficiently specified, including preprocessing steps, normalisation strategy, fold generation control, augmentation pipeline, model selection protocol, and precise inference configuration. Since the manuscript emphasises efficiency and accessibility, reproducibility should be one of its strongest features. The revised version should provide a substantially clearer experimental protocol and, if possible, an accessible code repository for review.

8. Interpretability claims are currently qualitative and selective

A notable strength of the paper is the attempt to provide interpretable token attention maps. However, the interpretability analysis remains largely qualitative and appears to rely on a small number of visual examples. This is not yet sufficient to support a strong interpretability claim. The authors should provide a more systematic evaluation of the attention maps, for example by analysing multiple cases, quantifying correspondence with lesion regions, or including expert assessment of whether the highlighted regions are stable and clinically meaningful. As it stands, the interpretability discussion is promising but preliminary.

9. Key design choices are fixed without sensitivity analysis

Several important architectural decisions appear to be fixed without adequate justification, most notably the number of learned tokens and the depth or width of the Transformer component. Because the central premise of the work is an efficiency-performance trade-off, these parameters should not remain unexplored. The revised manuscript would benefit substantially from a sensitivity study showing how token count and Transformer complexity affect memory usage, inference time, and segmentation quality. This would help establish whether the chosen configuration is principled or merely one reasonable setting among many.

10. Practical hardware claim should be moderated

The paper repeatedly emphasises operation on common or constrained hardware, yet the experiments were conducted on a workstation-class GPU with 24 GB memory. Although the relative efficiency improvements are important, the phrase common hardware may overstate the practical accessibility demonstrated by the current study. The authors should either moderate this language or provide additional evidence showing that the proposed framework remains viable under more modest hardware conditions, such as lower-memory GPUs or reduced deployment settings.

11. Discussion and conclusion occasionally overreach

The manuscript concludes with broad claims regarding the democratisation of foundation-model style methods for biomedical imaging. While this is an interesting future direction, the present experimental study does not yet establish such a claim. The discussion should more carefully distinguish between demonstrated findings, plausible interpretations, and future possibilities. A more disciplined conclusion would increase the credibility of the manuscript.

12. Language and presentation require editorial revision

The manuscript contains several wording issues, typographical errors, and minor presentation inconsistencies that reduce readability. In addition, some figure captions and explanatory statements would benefit from clearer and more self-contained phrasing. A careful language revision is recommended to improve precision, professionalism, and overall readability.

Recommendation to the editor

The manuscript has merit and addresses a relevant problem, but substantial revision is required before its scientific contribution can be properly assessed. I therefore recommend Major Revision. The revised version should strengthen the experimental benchmark, improve the fairness and transparency of the comparison protocol, expand the evaluation metrics, provide a more rigorous interpretability analysis, and align the stated contribution with the actual ablation evidence.

Reviewer #4: 1- Add the names of the datasets used in the abstract.

2- Explaining research contributions in clear and simple bullet points

3- Add a table summarizing the related work in terms of the name of the dataset used, the techniques and metrics adopted, as well as the advantages and disadvantages of each research paper.

4- Summarize the conclusions in a way that reflects only the most important findings of the researcher and proposed future work.

5- Add references for the period between 2025-2026, with no less than two references for each year.

**********

6. PLOS authors have the option to publish the peer review history of their article (what does this mean?). If published, this will include your full peer review and any attached files.

If you choose “no”, your identity will remain anonymous but your review may still be made public.

**Do you want your identity to be public for this peer review?**  For information about this choice, including consent withdrawal, please see our Privacy Policy.

Reviewer #1: No

Reviewer #2: **Yes:**  Shake Ibna Abir

Reviewer #3: No

Reviewer #4: No

**********

[NOTE: If reviewer comments were submitted as an attachment file, they will be attached to this email and accessible via the submission site. Please log into your account, locate the manuscript record, and check for the action link "View Attachments". If this link does not appear, there are no attachment files.]

To ensure your figures meet our technical requirements, please review our figure guidelines: https://journals.plos.org/plosone/s/figures

You may also use PLOS’s free figure tool, NAAS, to help you prepare publication quality figures: https://journals.plos.org/plosone/s/figures#loc-tools-for-figure-preparation.

NAAS will assess whether your figures meet our technical requirements by comparing each figure against our figure specifications.

# Author comment: Author response to Decision Letter 1

**DOI:** 10.1371/journal.pone.0354511.r003

2 Jul 2026

Original Manuscript ID: PONE-D-26-10976R1

Original Article Title: “TokenUNet: A New Case for Transformers Integration in Efficient and Interpretable 3D UNets for Brain Imaging Segmentation”

To: Editor and Reviewers

Re: Response to reviewers

Dear Editor,

Thank you very much for the very detailed analysis of the paper and for the extremely useful comments.

We answered each comment by the reviewers and we modified the text according to them.

In response to Reviewers' suggestions, we moved all our experimental analyses to the nnunetv2 framework, applying the very same 5 fold cross-validation protocol to all our proposed and competitor architectures, unifying hyperparameters at every level apart from the architecture peculiarities that define the different models.

This change alone, although computation heavy, solved many of the weak points of the previous version and in our opinion greatly elevates the robustness of our methods and the validity of discussions based on our results.

Importantly, we recorded different metrics and quantified questions regarding the interpretability of spatial attention maps created by TokenLearner and TokenFuser.

We hope our architectures and experimental infrastructure is now both more solid and clear than before the first round.

We deeply thank our reviewers for motivating these changes.

We are uploading (a) our point-by-point response to the reviewers’ comments (below), (b) an updated manuscript with yellow highlighting indicating changes, and (c) a clean updated manuscript without highlights.

Best regards,

Louis Fabrice Tshimanga and the Authors

Introductory response for Reviewer 1

Dear Reviewer 1,

Thank you for your commitment to our manuscript.

In the following you will find a response to the points you raised.

Best regards,

The Authors

Reviewer#1, Concern #1

“For the number of Tokens (N), the author only adopted a fixed value of N=8, lacking specific selection basis and failing to conduct parameter sensitivity analysis. It is necessary to supplement comparative experiments with different token numbers such as N=4, 8, 16, and 32 to illustrate the basis for the optimal setting and stability”

Author response: It is true that we adopted a fixed value, which we wanted to be minimal for computational reasons, and larger than the number of output labels for flexibility. The number of tokens N=8 is the minimal setting proposed in the original TokenLearner work, which also uses N=16. We agree that it is worthwhile to expand the experiments with different token numbers.

Author action: As a trade-off between exploration of different numbers, and exploitation of efficient settings, we decided to only experiment with N=8 and N=32.

Reviewer#1, Concern #2

“In Section 3.3, the author does not specify the data augmentation strategies, augmentation types, and corresponding parameters used during training.”

Author response: The reviewer is correct in noticing the missing specification. We agree that the augmentation settings are a key part of training and evaluation with regard to generalizations. We have now reframed our experiments into the nnUNet methodology, and we are detailing the augmentations performed in its recipe.

Author action: We have complemented the Method Section with descriptions of the spatial, noise and intensity data augmentations native to the nnunet framework, from line 278.

Reviewer#1, Concern #3

“The paper exhibits inconsistent tense usage when describing specific completed research activities, including model training, data processing, and result analysis. For concrete experimental operations, data processing procedures, model training setups, and the research results obtained in this study, the simple past tense should be used consistently.”

Author response: Thank you for highlighting this problem that may hinder readability of the paper.

Author action: We have harmonized the past tense of the manuscript for concrete procedures and results, specifically in the Method and Results Sections.

Introductory response for Reviewer 2

Dear Reviewer 2,

Thank you for your commitment to our manuscript.

In the following you will find a response to the points you raised.

Best regards,

The Authors

Reviewer#2, Concern #1

“While the use of TokenLearner is interesting, the manuscript does not sufficiently clarify how this contribution advances beyond existing token-reduction or efficient attention mechanisms. Moreover, recent developments in efficient Transformers, lightweight attention, and hybrid CNN-Transformer architectures are not thoroughly discussed. A stronger and more critical comparison with recent state-of-the-art approaches is necessary to convincingly establish the novelty and significance of the proposed method.”

Author response: Thank you for highlighting missing connections to extensive development in deep learning architectures. We have focused on readily available, torch-first, first principle approaches, compared for example to low-level development such as new CUDA kernels and hardware-friendly implementations of mathematical operations; on the architecture side, we have considered the developments that consistently occur in the medical imaging field, namely in the BraTS challenge, where for example the Swin-UNETR is a renowned hybrid CNN-Transformer architecture. However these different lenses were not extensively compared and discussed in the first version of our manuscript.

Author action: We extended the contextualization of efficiency-based approaches and developments related to our architectural approach in the Related Works subsection of the Introduction (from line 126 in particular).

Reviewer#2, Concerns #2, #4

“The experimental evaluation lacks sufficient rigor and breadth. Although the authors compare their method with UNet and SwinUNETR, the evaluation is limited to a single dataset (FeTS/BraTS subset) and a single metric (Dice score) . This is insufficient to demonstrate generalizability. Additional evaluation on other datasets or tasks would significantly strengthen the claims. Furthermore, no statistical significance testing is provided to support the reported performance differences (e.g., 87.21% vs 86.75% Dice), which are relatively small. Without statistical validation, it is unclear whether these improvements are meaningful or within variance.”

“The manuscript relies solely on Dice score for evaluation. While Dice is a standard metric for segmentation, it does not fully capture model performance in clinical settings. Metrics such as Hausdorff distance, sensitivity, specificity, or region-wise performance would provide a more comprehensive evaluation. Additionally, no discussion is provided regarding clinical significance or potential deployment implications, which is important given the biomedical application domain.”

Author response: Thank you for underlining the importance of statistical testing and multiple evaluation measures. We failed to express how we do not aim to achieve superiority on a single measures; rather we aimed to approximate parity within standard errors under reduced computation, which we managed. Nonetheless, we moved to full compatibility with the nnunetv2 protocol and now embrace the use of multiple performance measures, and follow the FeTS/BraTS example by reporting not only Dice score but also precision and recall, in order to highlight the models’ tendencies towards false positive and false negative segmentations. While the images cover aggregate measures, the code will open source and show all per-label results, adding IoU, TP, TN, FP, FN. Moreover, we have discussed the implications of these aspects in translational, clinical domains.

Finally, we agree that using more datasets would strengthen the claims of generalizability, however there is a lack of suitable multimodal datasets for brain segmentation, and thus we leave the extension of such experiments to future research, while discussing explicitly their importance.

Author action: We expanded the set of performance measures, highlighted where performance differences are higher than sample variance, and discussed the implications in Results Fig 5 and 6 in particular. We have expanded on translational limits in the Limitations subsection in the Discussion Section, from line 417.

Reviewer#2, Concern #3

“A critical issue is the lack of fair comparison across models. The manuscript explicitly states that hyperparameter tuning was avoided to reduce computational cost. While this aligns with the goal of efficiency, it raises concerns about whether competing models (especially SwinUNETR) were optimally configured. Differences in optimization strategies (SGD vs AdamW, different learning rates) further complicate fair comparison . This may bias the results in favor of the proposed model. A fair comparison requires consistent tuning protocols or justification that all models are equally optimized.”

Author response: Thank you for raising the point. While we tried to use the parameters suggested in the papers presenting each model class, it is true that the set up makes it hard to evaluate whether the comparison in itself is fair, because the degrees of freedom are growing exponentially.

For this reason we have revised our experimental battery in order to use the nnUNet suggested defaults for all model classes, thus evaluating fairly which models are the best for these widely accepted settings.

Author action: We moved our evaluations to a battery of models implemented in nnUNet cross validations with default identical hyperparameters.

Reviewer#2, Concern #5

“Although the authors mention that code will be released upon publication, reproducibility remains limited in the current submission. Important details such as hyperparameter sensitivity, initialization variability, and robustness across different training conditions are not discussed. Moreover, the use of batch size 1 with gradient accumulation and specific architectural simplifications may impact reproducibility across different hardware environments.”

Author response: We agree that the first submission, as noted in other reviewers’ concern, was missing key information to reproduce unambiguously our results. We have now both enhanced the clarity of descriptions in our pipeline, as well as published a repository to actually inspect and run the analyses coming with the reviewed manuscript. Regarding the use of gradient accumulation and batch size=1, we avoid all related problems by adopting the nnUNet framework..

Author action: We are publishing code for the revision, we described augmentations and performance measures in the manuscript, and highlighted the potential differences of running such experiments on different GPUs in the Limitation subsection.

Reviewer#2, Concern #6

“The manuscript occasionally overstates its contributions. For example, the claim that the model “tops” SwinUNETR performance is based on a relatively small improvement in Dice score, which may not be statistically significant. Similarly, claims regarding interpretability through attention maps are not quantitatively evaluated. While visualizations (e.g., attention maps in Figure 7) are useful, they remain qualitative and require more rigorous validation.”

Author response: The reviewer is correct in that the model does not “top” SwinUNETR in pure performance. The correct statement is that our model on average reached SwinUNETR’s performance, for a fraction of the cost, topping it in terms of efficiency. Regarding interpretability, while explainable and interpretable AI are subdomains of their own, with proper quantification of what is meant by “explainable” and “interpretable”, our models are only qualitatively interpretable, but inherently so, with the important addition that attention maps are a priori mechanistically responsible for the computations and decisions of our model, because they dictate which voxels are sampled and at what strength, whereas attention maps based on gradients such as Grad-CAM are post-hoc interpretation tools.

Author action: We have elucidated the differences between interpretability of TokenLearner attention maps, Grad-CAM-like methods, with pointers to the domains of explainability and interpretability research in Related Works as well as Discussion Section, Limitation subsection (lines 150 and 435).

Introductory response for Reviewer 3

Dear Reviewer 3,

Thank you for your commitment to our manuscript.

In the following you will find a response to the points you raised.

Best regards,

The Authors

Reviewer#3, Concern #1

“The manuscript positions Token-UNet primarily as a new and efficient case for Transformer integration. However, the ablation narrative indicates that the largest performance gain is obtained from the transition from the classic UNet baseline to the modified UNet** backbone, while the Transformer itself appears to contribute less than the TokenLearner and TokenFuser bottleneck. This is a crucial point because it affects how the novelty should be framed. The authors should revise the title, abstract, introduction, and conclusion so that the stated contribution is fully aligned with the experimental evidence. It should be made explicit whether the main innovation is the token bottleneck design, the revised additive UNet backbone, the efficient insertion of a lightweight Transformer, or the combined architectural pipeline.”

Author response: The reviewer is right in addressing the possible confusion in our contributions and their relevance to the field.

First of all, we clarify that our aim is not that of achieving state-of-art results in performance metrics.

Our contributions should be framed as such, building the literal “case” that can box Transformer-like models in an efficient way, and pushing forward the case for doing so:

1) the UNet backbone is reinforced in order to comply with the goals of small memory and time complexity/occupation, modularity, and the original aim of UNets in general;

2) the token bottleneck is a stand-alone means to implement an effective information bottleneck with interpretable characteristics, as well as an established tokenization alternative that we suggest should be adopted widespread when considering hybrid CNN-Transformer architectures.

The addition of a basic Transformer is a proof of concept that the tokenization scheme is effective and makes such Transformers more efficient than standard tokenization by voxel patches.

We leave to future works to fully demonstrate the advantage of pretraining and fine-tuning large Transformers, since in other domains it has had the largest returns.

Meanwhile we highlight a strong case for doing so efficiently.

Author action: We have better framed the Introduction of our main contribution, as well as the discussion of our future objectives and suggestions for the research community to expand on these results, in the Future directions subsection of our Discussions.

Reviewer#3, Concern #2, #3, #4, #5

“Insufficient baseline breadth for a strong segmentation claim.

Fairness of the optimisation and training protocol.

Evaluation metrics are too narrow for a medical segmentation study.

Statistical analysis should be strengthened.”

Author response: We agree with the reviewer that the best generalist baseline is the nnU-Net framework. We have thus decided to rerun our pipeline with both its baseline 3D architecture, as well as our previous comparative baselines.

Author action: We completely renovated both the Methods and the Result section in order to implement the reviewer suggestion and make a strong case for our recalibrated claims. We evaluate 5-fold cross validations of several models using the same hyperparameters and training recipe, identifying a variant that performs statistically better, which is a target for further developments with personalized hyperparameters.

Reviewer#3, Concern #6

“All experiments are conducted on a single dataset under internal five-fold cross-validation. While this is a reasonable starting point, the manuscript occasionally generalises its conclusions to 3D biomedical imaging more broadly. At present, the evidence supports only a more limited conclusion tied to this specific tumour segmentation setting. The

Attachment Submitted filename: TokenUNet.docx-2.pdf

# Peer review: Decision Letter 1

**DOI:** 10.1371/journal.pone.0354511.r004

**Authors:** Kumaradevan Punithakumar

10 Jul 2026

TokenUNet: A New Case for Transformers Integration in Efficient and Interpretable 3D UNets for Brain Imaging Segmentation

PONE-D-26-10976R1

Dear Dr. Tshimanga,

We’re pleased to inform you that your manuscript has been judged scientifically suitable for publication and will be formally accepted for publication once it meets all outstanding technical requirements.

Within one week, you’ll receive an e-mail detailing the required amendments. When these have been addressed, you’ll receive a formal acceptance letter and your manuscript will be scheduled for publication.

An invoice will be generated when your article is formally accepted. Please note, if your institution has a publishing partnership with PLOS and your article meets the relevant criteria, all or part of your publication costs will be covered. Please make sure your user information is up-to-date by logging into Editorial Manager at Editorial Manager® and clicking the ‘Update My Information' link at the top of the page. For questions related to billing, please contact billing support.

If your institution or institutions have a press office, please notify them about your upcoming paper to help maximize its impact. If they’ll be preparing press materials, please inform our press team as soon as possible -- no later than 48 hours after receiving the formal acceptance. Your manuscript will remain under strict press embargo until 2 pm Eastern Time on the date of publication. For more information, please contact onepress@plos.org.

Kind regards,

Kumaradevan Punithakumar

Academic Editor

PLOS One

Additional Editor Comments (optional):

Reviewers' comments:

Reviewer's Responses to Questions

**Comments to the Author**

1. If the authors have adequately addressed your comments raised in a previous round of review and you feel that this manuscript is now acceptable for publication, you may indicate that here to bypass the “Comments to the Author” section, enter your conflict of interest statement in the “Confidential to Editor” section, and submit your "Accept" recommendation.

Reviewer #1: All comments have been addressed

Reviewer #3: All comments have been addressed

Reviewer #4: (No Response)

**********

2. Is the manuscript technically sound, and do the data support the conclusions?

The manuscript must describe a technically sound piece of scientific research with data that supports the conclusions. Experiments must have been conducted rigorously, with appropriate controls, replication, and sample sizes. The conclusions must be drawn appropriately based on the data presented.

Reviewer #1: Yes

Reviewer #3: Yes

Reviewer #4: (No Response)

**********

3. Has the statistical analysis been performed appropriately and rigorously?

Reviewer #1: Yes

Reviewer #3: Yes

Reviewer #4: (No Response)

**********

4. Have the authors made all data underlying the findings in their manuscript fully available?

The PLOS Data policy requires authors to make all data underlying the findings described in their manuscript fully available without restriction, with rare exception (please refer to the Data Availability Statement in the manuscript PDF file). The data should be provided as part of the manuscript or its supporting information, or deposited to a public repository. For example, in addition to summary statistics, the data points behind means, medians and variance measures should be available. If there are restrictions on publicly sharing data—e.g. participant privacy or use of data from a third party—those must be specified.

Reviewer #1: Yes

Reviewer #3: Yes

Reviewer #4: (No Response)

**********

5. Is the manuscript presented in an intelligible fashion and written in standard English?

PLOS ONE does not copyedit accepted manuscripts, so the language in submitted articles must be clear, correct, and unambiguous. Any typographical or grammatical errors should be corrected at revision, so please note any specific errors here.

Reviewer #1: Yes

Reviewer #3: Yes

Reviewer #4: (No Response)

**********

6. Review Comments to the Author

Please use the space provided to explain your answers to the questions above. You may also include additional comments for the author, including concerns about dual publication, research ethics, or publication ethics. (Please upload your review as an attachment if it exceeds 20,000 characters)

Reviewer #1: I am satisfied that the authors have fully and individually addressed my original three concerns; I have no remaining revision suggestions.

Reviewer #3: The authors are commended for their meticulous, point-by-point response to the previous evaluation. All concerns, methodological ambiguities, and structural recommendations raised during the initial review cycle have been thoroughly addressed. The manuscript has been significantly improved in terms of technical clarity, presentation flow, and experimental depth. Incorporating the requested comparative analyses and validation metrics has strengthened the core claims and solidified the overall contribution of the proposed framework. The updated figures and text now provide an excellent, reproducible narrative. Having fully completed the required revisions to a high standard, the paper is in an acceptable form and recommended for publication.

Reviewer #4: (No Response)

**********

7. PLOS authors have the option to publish the peer review history of their article (what does this mean?). If published, this will include your full peer review and any attached files.

If you choose “no”, your identity will remain anonymous but your review may still be made public.

**Do you want your identity to be public for this peer review?**  For information about this choice, including consent withdrawal, please see our Privacy Policy.

Reviewer #1: No

Reviewer #3: **Yes:**  Dr Yasir Abdullah R

Reviewer #4: No

**********

# Editor report: Acceptance letter

**DOI:** 10.1371/journal.pone.0354511.r005

**Authors:** Kumaradevan Punithakumar

PONE-D-26-10976R1

PLOS One

Dear Dr. Tshimanga,

I'm pleased to inform you that your manuscript has been deemed suitable for publication in PLOS One. Congratulations! Your manuscript is now being handed over to our production team.

At this stage, our production department will prepare your paper for publication. This includes ensuring the following:

* All references, tables, and figures are properly cited

* All relevant supporting information is included in the manuscript submission,

* There are no issues that prevent the paper from being properly typeset

You will receive further instructions from the production team, including instructions on how to review your proof when it is ready. Please keep in mind that we are working through a large volume of accepted articles, so please give us a few days to review your paper and let you know the next and final steps.

Lastly, if your institution or institutions have a press office, please let them know about your upcoming paper now to help maximize its impact. If they'll be preparing press materials, please inform our press team within the next 48 hours. Your manuscript will remain under strict press embargo until 2 pm Eastern Time on the date of publication. For more information, please contact onepress@plos.org.

You will receive an invoice from PLOS for your publication fee after your manuscript has reached the completed accept phase. If you receive an email requesting payment before acceptance or for any other service, this may be a phishing scheme. Learn how to identify phishing emails and protect your accounts at https://explore.plos.org/phishing.

If we can help with anything else, please email us at customercare@plos.org.

Thank you for submitting your work to PLOS ONE and supporting open access.

Kind regards,

PLOS ONE Editorial Office Staff

on behalf of

Professor Kumaradevan Punithakumar

Academic Editor

PLOS One
